# Hominin brain size increase has emerged from within-species encephalization

**DOI:** 10.1073/pnas.2409542121

**Published:** 2024-11-26

**Authors:** Thomas A. Püschel, Samuel L. Nicholson, Joanna Baker, Robert A. Barton, Chris Venditti

**Affiliations:** ^a^Institute of Human Sciences, School of Anthropology and Museum Ethnography, University of Oxford, Oxford OX2 6PE, United Kingdom; ^b^School of Biological Sciences, University of Reading, Reading RG6 6AS, United Kingdom; ^c^Climate Geochemistry, Max Planck Institute for Chemistry, Mainz 55128, Germany; ^d^Department of Anthropology, Evolutionary Anthropology Research Group, University of Durham, Durham DH1 3LE, United Kingdom

**Keywords:** encephalization, hominins, phylogenetic comparative methods

## Abstract

The fact that rapid brain size increase was clearly a key aspect of human evolution has prompted many studies focusing on this phenomenon, and many suggestions as to the underlying evolutionary patterns and processes. No study to date has however separated out the contributions of change through time within vs. between hominin species while simultaneously incorporating effects of body size. Using a phylogenetic approach never applied before to paleoanthropological data, we show that relative brain size increase across ~7 My of hominin evolution arose from increases within individual species which account for an observed overall increase in relative brain size. Variation among species in brain size after accounting for this effect is associated with body mass differences but not time. In addition, our analysis also reveals that the within-species trend escalated in more recent lineages, implying an overall pattern of accelerating relative brain size increase through time.

## Significance

Our study significantly advances our comprehension of human brain evolution by employing a unique approach to dissect changes in brain size throughout the complete fossil record of hominins. By disentangling the dynamics of brain size change that occur within species from those occurring across species, we unveil that increases in brain size primarily occurred within the lineages comprising a single species. Such a pattern gives rise to the overall brain expansion that scientists herald as a trademark of modern humanity. Furthermore, we reveal a trend of accelerating brain size growth in more recent lineages. This nuanced understanding deepens our insight into the evolutionary trajectory of human cognition and behavior, crucial for unraveling the complexities of our species’ unique traits.

One of the most evident evolutionary changes during human evolution and one intimately associated with our unique cognitive and behavioral traits has been an increase in brain size (1–5). Encephalization (i.e., relative brain size increase) during human evolution has long been debated, and several studies have compared hominin cranial capacities across species to propose possible adaptive mechanisms acting upon brain size variation among hominins (5–10). Some have argued for gradual growth over time (6, 11, 12), while others propose punctuated equilibrium with rapid increases followed by stasis (13–16). Other studies support a combination of both models (7, 17, 18), while others claim they cannot be distinguished (19). These contradictory views arise in part from conflating distinct phenomena (8, 20, 21); namely, the role of speciation events on trait diversification (anagenesis vs. cladogenesis), and the relative importance of gradual vs. pulsed evolution (i.e., different aspects of punctuated equilibrium) (20). One previous study also emphasized a need to partition hominin brain size evolution in order to assess change within lineages (i.e., phyletic change) and between lineages (i.e., cladogenesis or lineage extinction), as such an approach could provide a more detailed evaluation for the evolution of this trait (8).

Variation within-species is also an important consideration in understanding macroevolutionary patterns in encephalization, as well as in absolute brain size, and as such, other researchers have focused on both relative and absolute brain size change at the intraspecific level. These studies are mostly limited to those species in which their hypodigm is large enough to enable meaningful analyses (e.g., *Homo sapiens, Homo neanderthalensis, Homo heidelbergensis, Homo erectus, Homo habilis, Paranthropus boisei, Australopithecus afarensis*) (17, 22–29). Inconsistent results have even been found for several species, with some reporting that there is no evident trend through time (10, 24, 29), while others seem to point toward an increase (17, 26–28), and yet others report apparent reductions (30). Inconsistent results have even been shown for the same species (24, 28). These conflicting results hint at complex patterns not fully captured by approaches used previously. Simultaneously analyzing within- and between-species variation in brain size while accounting for body size has the potential to provide a more comprehensive and nuanced understanding of hominin brain evolution. Here, we present a comprehensive set of analyses that allow us to study relative cranial capacity evolution through time, by explicitly considering a) body mass, b) within- and between-species variability, and c) phylogenetic relatedness and uncertainty.

**Fig. 1.**
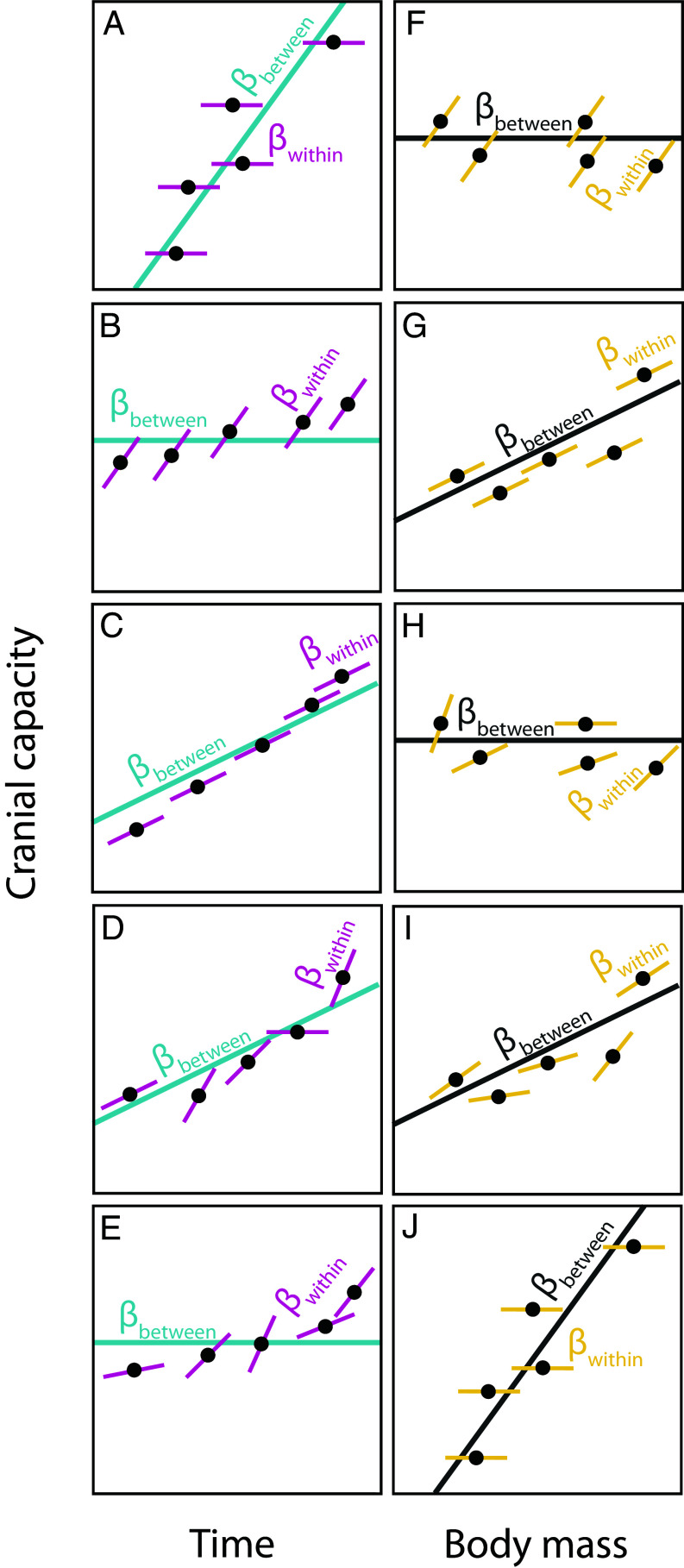
Schematic representation of different scenarios reflecting how both body size and time can show different effects both within and between species. We show five different scenarios for how within- and between-species effects can differ for time resulting in an increased cranial capacity (*A*–*E*). These scenarios can occur together with different within- and between-species body mass patterns (*F*–*J*). The schematic figure shows the within-subject slopes (thinner solid lines; β_within_) of five hypothetical species with the corresponding between-species slope (thicker solid lines; β_between_). Please note that the data points shown in this figure are not truly independent as they are correlated according to a phylogenetic correlation matrix.

Fig. 1 illustrates the different evolutionary scenarios that our analyses enable us to distinguish between. First, a between-species brain size increase correlated with time but not within-species (Fig. 1*A*). Second, a within-species brain increment associated with time but no evident trend between species (Fig. 1*B*). Third, a combined between- and within-species trend toward increasing brain size (Fig. 1*C*). Fourth, a scenario where later species exhibited larger cranial capacities, and where each lineage could show its own within-species trend that could be horizontal, positive, or negative (Fig. 1*D*). Fifth, variable within-species encephalization patterns with no between-species trend (Fig. 1*E*). Any of these brain size increase scenarios can occur together with different within- and between-species body mass relationships (Fig. 1 *F–J*) and our analyses can reveal which of these alternative scenarios is most consistent with the data.

**Fig. 2.**
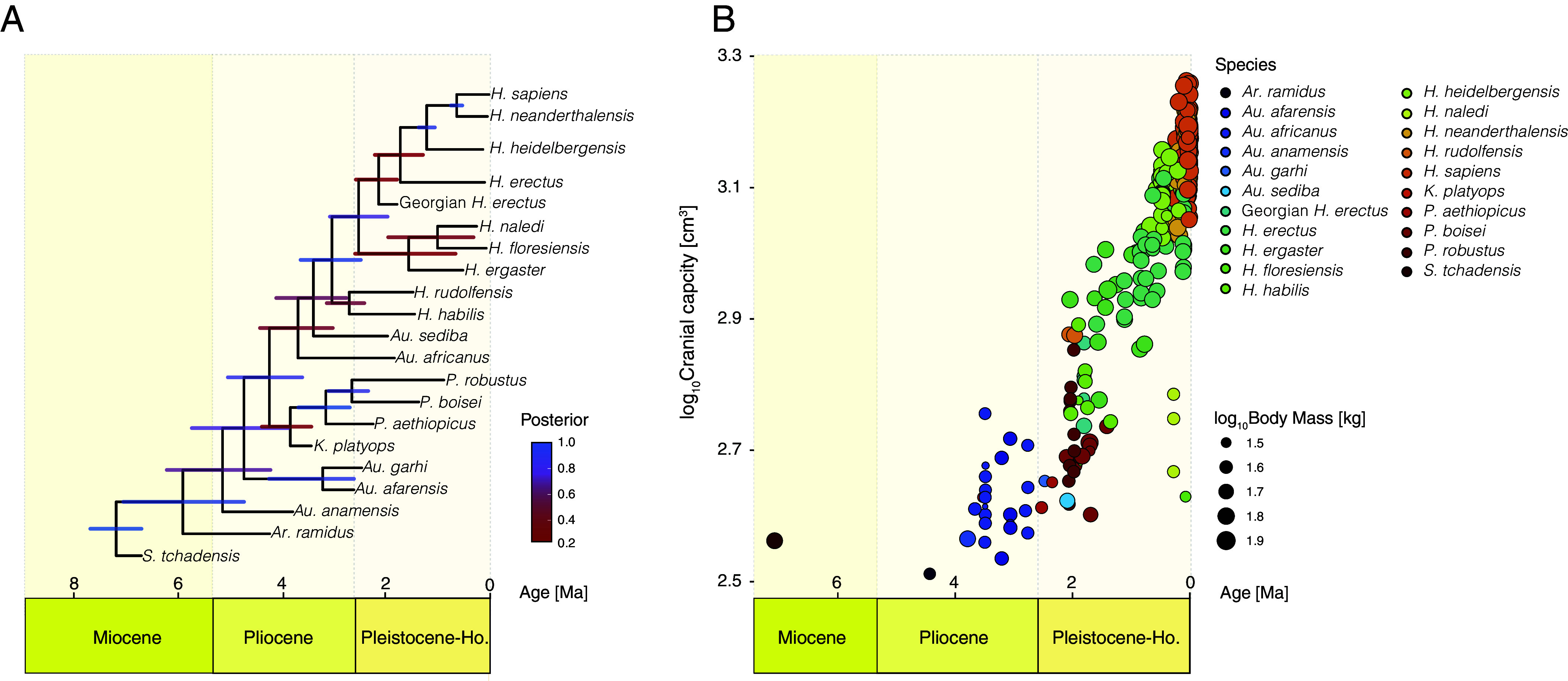
A graphical representation of the phylogenetic trees and datasets used in our analyses. (*A*) Maximum a posteriori (MAP) tree summarizing the posterior sample of 60,000 hominin phylogenies obtained from our combined-evidence Bayesian phylogenetic analysis. The length of the bars on the MAP tree corresponds to the age 95% highest posterior density interval (HPD), while the color represents posterior support. Please note that gorilla, chimpanzee, and Denisovan were removed from these 1,000 phylogenies; (*B*) Hominin cranial capacity and body mass through time. The values from this figure correspond to the mean values of the 1,000 datasets used in this study.

We first undertook a “combined-evidence” Bayesian phylogenetic reconstruction of hominin species (31, 32) using stratigraphic, molecular, and morphological data to account for shared ancestry and its associated uncertainty in our comparative analyses (Fig. 2*A*, *Materials and Methods*, and *SI Appendix*, Fig. S1 and Table S1). We obtained a posterior sample of phylogenetic trees from which we randomly sampled 1,000. We then removed the gorilla, chimpanzee, and Denisovan prior to our subsequent analyses (i.e., restricting analyses to hominins for which cranial capacity data are available). Such an approach has the advantage of incorporating uncertainty about hominin relationships: Rather than assuming a single supposedly correct hominin phylogeny, we were able to test our models using a sample of phylogenetic trees showing different topologies. We compiled the largest extinct hominin dataset to date comprising cranial capacity (n = 285), body mass (n = 431), and chronometric age data for multiple specimens representing the species in our phylogenies (Fig. 2*B*) (*Materials and Methods*). We associated every specimen with cranial capacity with a species category, body mass value, and chronometric age using well-defined criteria (*Materials and Methods*) which allowed us to consider uncertainties related to taxonomic assignment, body mass estimates, and temporal range (an alternative phylogenetic imputation procedure was also tested but yielded qualitatively identical results; *Materials and Methods*). This process was repeated 1,000 times resulting in 1,000 unique datasets, each one of them comprising the same 285 individuals with cranial capacities but now with associated body mass and chronometric age values, as well as a taxonomic label. This means that our 1,000 datasets contain samples of both phylogenetic trees and hominin data, and as such incorporate the uncertainty in both. One of the main advantages of such an approach is that it enables testing different modeling scenarios more easily, thus facilitating effective assessment of diverse modeling decisions, including alternative taxonomic classifications. Therefore, we decided to robustly assess alternative modeling assumptions by repeating the above procedures with additional modeling sets (See *SI Appendix,* Table S2 for a complete list of the 24 tested models, as well as associated numerical results; please note that each of the models was run 1,000 times using our 1,000 datasets and 1,000 phylogenies). This modeling approach represents, to our knowledge, the most comprehensive attempt to incorporate uncertainty and assess modeling decisions ever undertaken in the study of encephalization. Furthermore, as our data are available here, other alternative scenarios can naturally be envisioned and easily tested by informed readers using our proposed approach. We acknowledge that additional uncertainty sources exist, but we have tried to incorporate as many as possible, as well as being explicit about them and their associated assumptions.

We used Bayesian phylogenetic generalized linear mixed models (PGLMM) (33, 34) to test the relationship between cranial capacity relative to body mass and time at both the intra- and interspecific levels. We take advantage of a “within-group centering” (35) approach (*Materials and Methods*) to study the relative effects of within-species and between-species variation. This procedure resulted in four different variables: the between-species body mass and time effects and the within-species body mass and time cofactors. We allowed slopes and intercepts to vary within-species for time (Model 1), as well as for time and body mass (Model 2) to account for potential species-specific differences. To distinguish between an anagenetic vs. a cladogenetic scenario of brain size evolution, we repeated Model 1 but also including an additional covariate (i.e., log_10_ node count) that can be regarded as a speciation rate metric obtained by counting the number of nodes between the root and each tip present in the phylogeny (Model 3). We also ran an additional set of models including an interaction term between the between- and within-time variables to assess whether brain size evolution accelerated through time (Model 4). Table 1 provides the definition of all our main models. We repeated every one of these modeling procedures 1,000 times using our 1,000 datasets and 1,000 phylogenies to ensure our results are robust to the various sources of uncertainty. We considered an effect significant if the obtained p Markov-chain Monte Carlo (pMCMC) values (36) were less than or equal to 0.05 in 95% of the 1,000 analyses carried out for each one of our modeling scenarios (Models 1 to 4). Rather than choosing one single model, these four alternatives are reported as all of them provide complementary aspects about relative brain size increase in hominin evolution such as the potential role of cladogenesis or whether there is an accelerating trend in relative brain size increase.

**Table 1.** Definitions and metrics for the four main models; random effects are in parentheses

Model	Description	Definition	DIC	*h* ^2^	Marginal R^2^	Conditional R^2^
M1	Model with varying slopes and intercepts for within-time effects	CC ∼ bm_between_ + bm_within_ + time_between_ + time_within_+ (phylogeny) + (species:time_within_)	−938.718	0.952	0.612	0.935
M2	Model with varying slopes and intercepts for within-body mass and within-time effects	CC ∼ bm_between_ + bm_within_ + time_between_ + time_within_+ (phylogeny) + (species:bm_within_) + (species:time_within_)	−953.615	0.888	0.61	0.947
M3	Model used to assess cladogenesis vs. anagenesis	CC ∼ bm_between_ + bm_within_ + time_between_ + time_within_+ NC + (phylogeny) + (species:time_within_)	−938.943	0.951	0.641	0.939
M4	Model used to test for accelerating evolution	CC ∼ bm_between_ + bm_within_ + time_between_ + time_within_ + time_between_:time_within_ + (phylogeny)	−888.397	0.92	0.615	0.882

## Results

**Fig. 3.**
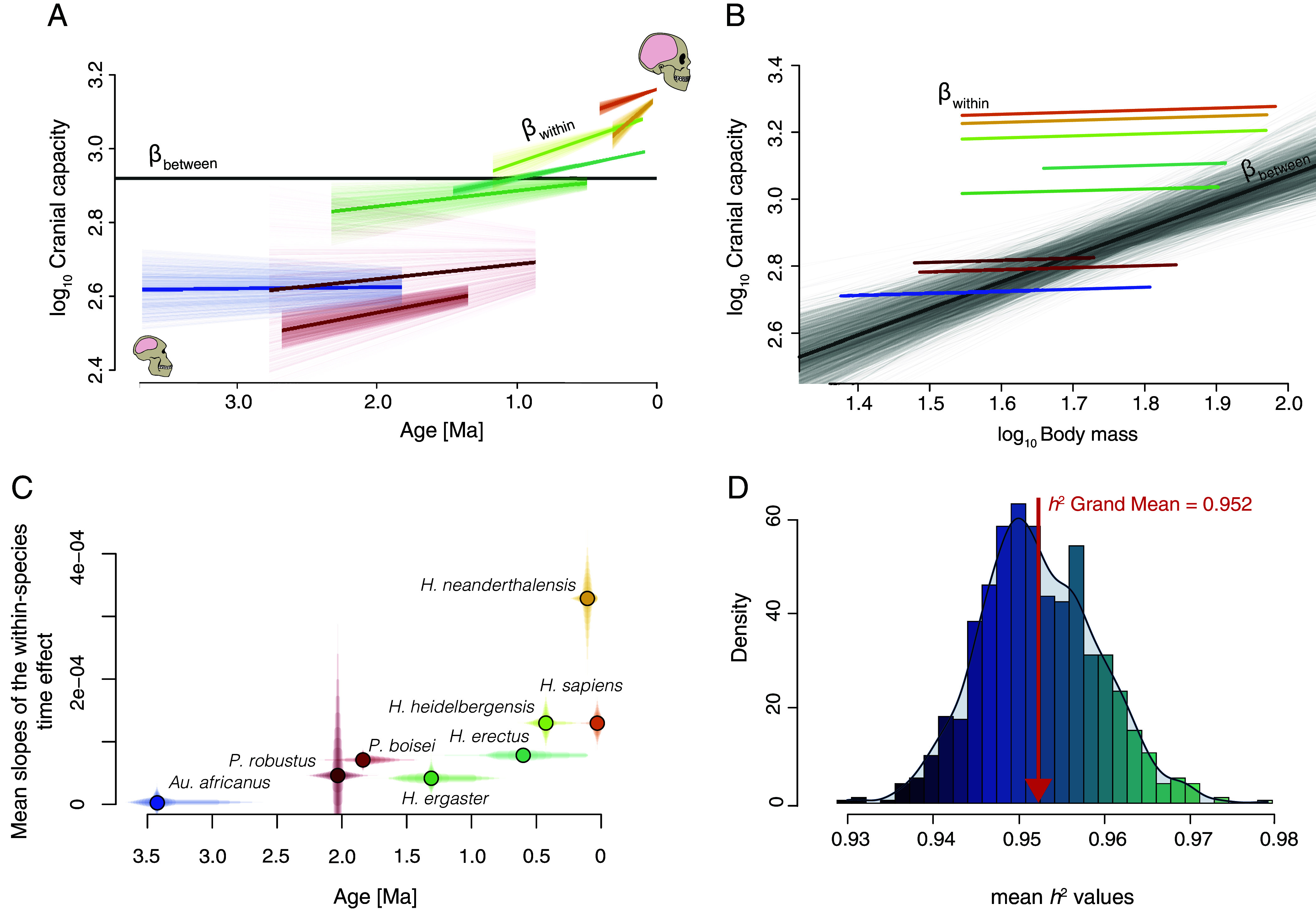
The relationship between cranial capacity, body size, and time both within- and between-species. (*A*) Within-species trends in cranial capacities through time. Individual lines are the mean predictions from 1,000 regressions of log_10_ cranial capacity on the log_10_ within-species time predictor. The length of these lines corresponds to the temporal span of each species; (*B*) 1,000 regression lines for the estimated between-species (gray lines) and the mean of the 1,000 within-species (color lines) for the body mass effect on cranial capacity. The darkest gray line represents to the mean value for the between-species regression lines. The length of the within-species regression lines corresponds to the body mass range for each species; (*C*) Mean slopes of the within-species time effect through time. The y-axis corresponds to the 95% quantile of the 1,000 within-species time effect slopes. Note that these values are positive here to facilitate interpretation through time. The *x*-axis is the 95% quantile of the randomly sampled ages per species obtained for every analyzed specimen from their specific temporal range; (*D*) distribution of the 1,000 mean values for *h^2^*, ranging from lower (darker blue) to higher (cyan) values. In *A*–*C* only those species with more than ten specimens in our dataset are depicted, although all parameters in all models are calculated using all species (see *Methods*).

Our results (Table 1 and *SI Appendix,* Tables S2–S5) show a strong and significant association between cranial capacity and between-species body mass. However, no association with body mass was found within species. We found no association between cranial capacity and between-species time differences, but there was a significant relationship at the intraspecific level for time (Fig. 3). Furthermore, even if there was a significant between-species time effect, its slope would be shallow as observed in all our models (*SI Appendix,* Table S4). As we found that there was no significant within-species body mass effect in our models, we report the results from Model 1 below (i.e., our model with a species-specific random effect for the within-species time variable), but all our models show qualitatively identical results (Table 1). Phylogenetic signal as measured by Lynch’s heritability (37) was close to one (*h*^2^ grand mean: 0.95) (Fig. 3*D*), highlighting the importance of considering shared ancestry when studying hominin brain data. R^2^ values [both marginal and conditional (38)] were consistently high (marginal R^2^ grand mean: 0.61; conditional R^2^ grand mean: 0.94), thus indicating the overall good fit of our models (*SI Appendix,* Table S3). Our results correspond to the expectations shown in Fig. 1 *E* and *J* in which there a is significant within-species slope variation for cranial capacity, with no significant effect at the between-species level with respect to time—along with no within-species slope variation and a significant between-species effect of body size. Later specimens exhibit larger cranial capacities as compared to earlier ones, but there is no evident trend at the interspecific level, together with a body mass between-species effect and no evident body mass–related trend within-species (Fig. 3 *A* and *B* and *SI Appendix,* Tables S4 and S5). It is important to keep in mind that our models are multiple regressions comprising both time and body mass between- and within-species effects, and as such the results need to be considered in tandem and not isolation. This means that cranial capacity increases within species and these increases persevere through time as there is a between-species body mass effect that allows cranial capacity to pick up where it left without a “reset” in terms of relative brain size. In other words, relative brain size increases along the branches of the phylogeny, and then the achieved relative brain size increments in every lineage persists through time owing to body mass increases at speciation points. It is important to bear in mind when interpreting our results that we are estimating the association between variables along the branches of a phylogenetic tree (i.e., evolutionary regression coefficients) (39). These coefficients indicate in our case how cranial capacity evolves along the branches of a phylogenetic tree as function of changes in other traits (i.e., within and between-species body mass and time), thus allowing us to recover the historical pattern of evolutionary change in cranial capacity.

Our findings illustrate the multilevel nature of hominin brain size evolution. Part of the contradictory observations made by previous studies are the result of not accounting for intra- vs. interspecies differences, as well as ignoring allometric and phylogenetic effects, as shown by the significant between-species body mass effect (Fig. 3*B*) and high phylogenetic signal (*h*^2^) values (Fig. 3*D*). Our modeling approach highlights the fundamental importance of disentangling intra- and interspecific levels of variation when analyzing brain size evolution, as not accounting for these differences results in observing an opposite encephalization pattern (*SI Appendix,* Table S4). A misleading result would also be obtained if a standard phylogenetic generalized least squares regression is applied, as such an approach would intrinsically conflate within- and between-species effects (*SI Appendix,* Table S4).

## Discussion

Our models show a significant within-species time effect, where slopes differ at the intraspecific level. Hence, hominin encephalization cannot simply be characterized by a common and shared temporal trend across all hominin species, challenging the notion that brain evolution has been consistently driven by a single long-term, global, and constant low-level directional selective pressure (6). Instead, we observe a positive association between relative cranial capacity and time within species, indicating temporal trends within each one of the analyzed species (Fig. 3*A*). This suggests different intensities of selection toward larger brain sizes within each hominin species during different time periods (Fig. 3*A*). For example, our analyses (Model 4) reveal that the within-species trend escalated in more recent lineages, implying an overall pattern of gradual but accelerating relative brain size increase through time (Fig. 3*C* and *SI Appendix,* Table S4). Early hominins such as *Au. africanus* have a shallow slope between cranial capacity and time (Fig. 3*C*), and our models suggest that other early species like *Au. afarensis*, while too small to visualize independently, are likely to have shown similar patterns. On the other hand, later species (e.g., *H. heidelbergensis* or *H. sapiens*) increased their brain sizes at a faster pace. The correlation between this within-species temporal trend (i.e., slopes of the within-species time effect) and time is strong (Pearson’s r: 0.74; Pearson’s r without *H. neanderthalensis*: 0.9). *H. neanderthalensis* show the fastest increase in brain size through time. This may be owing to the greater degree of encephalization reported in late Neanderthals as compared to earlier members of this species. This result challenges the antiquated notion of Neanderthals as a uniform species unable to respond quickly to their changing environments (40).

Our results also show that there is a strong association between brain size increase and body mass (Fig. 3*B*), which is consistent with evidence that suggests brain size and body size have not independently evolved over evolutionary time (41). In multiple primate species (e.g., macaques, baboons, apes), as well as *H. sapiens,* there is a low correlation between brain size and body mass at the intraspecific level even in very large samples (41–45). Our results, showing no significant relationship between brain size and body mass within species, generalize these assessments to all hominin species. This finding is consistent with the seminal study by ref. 46 that found low intraspecific and high interspecific phenotypic correlations when studying brain size:body size relationships. In addition, the results from Model 3, which assessed the role of anagenesis vs. cladogenesis on encephalization, show that while speciation events are not significant predictors of relative cranial capacity, there is still a within-species age effect, which is consistent with an anagenetic pattern of brain size evolution. This is further confirmed by the results of an additional model excluding the within- and between-time effects in which node count was still nonsignificant (*SI Appendix,* Tables S2–S5).

Taken together, our results show that there was a within-species increase in brain size during human evolution and that this pattern explains the overall increase in relative brain size across human evolution. This means that relative brain size macroevolution in hominins seems to be entirely explained by microevolutionary, population-level processes. This process is consistent with an anagenetic pattern as further shown by our results considering speciation events (Model 3). Traditionally, a gradual trend has been understood as the consequence of consistent directional selection at the within-species (i.e., population) level for larger brains represented by a single common slope (Fig. 1*B*). However, here we show that a similar pattern can also arise when different species display their own intraspecific increasing trends (Figs. 1*E* and 3*B*). This contradicts conventional punctuated equilibrium views that regard encephalization as the result of brief episodes of rapid increase separated by extended periods of stasis (13–15). The absence of a between-species time effect does not mean that there are no differences between species, but rather that there is a positive association between relative cranial capacity and time within-species (Fig. 3*A*), in which brain size increases within each lineage through time. This means that intraspecific increments in cranial capacity persist over time as result of brain size tracking interspecific changes in body mass. Our results are also consistent with an accelerating within-species increase through time (Model 4), in line with hypotheses that evoke a coevolutionary positive feedback process such as between brain size and sociality, culture, technology, or language (47–51).

While some previous studies have reported an accelerating pattern in the rates of evolution of brain size across hominins at the species level (12, 16, 52), our study is the first to have identified an accelerated increase in the allometric relationship between brain and body size within species through time, providing an intraspecific mechanistic explanation for previously reported results across species. Overall, our results show the multilevel aspects of human brain expansion, as well as the need for future studies to incorporate this hierarchical complexity. Our methods offer an effective quantitative framework to study within- and between-species trait evolution, and as such open an avenue of research testing explicit hypotheses about which factors underly intra- and interspecific brain expansion during hominin evolution. A logical extension of the applied approach is to incorporate additional predictor variables, such as environmental and/or climatic factors, which could allow researchers to test longstanding hypotheses about the potential effect of climate on encephalization.

## Materials and Methods

### Phylogenetic Analyses.

To infer hominin phylogenetic relationships, we carried out a combined-evidence Bayesian phylogenetic analysis of extant and fossil hominin species, combining morphological and molecular data as well as stratigraphic range data (first and last occurrences from the fossil record e.g., refs. 53–55, Dataset S1), a “Fossilized Birth Death Range Process” (56) implemented within RevBayes v.1.1.0 (57). The morphological data came from ref. 58 and comprised a supermatrix of 391 craniodental characters. We removed *H. antecessor* from this matrix as it corresponds to a single juvenile individual with mostly missing data in the original dataset (58). We also added additional morphological characters that were originally coded as missing in two species (i.e., *Au. anamensis* and *H. floresiensis*) using information from refs. 59 and 60 (Dataset S2). The Mkv+Γ model (61) was used for the morphological data, partitioned by number of states and ordered or unordered characters. Possible ascertainment bias in the morphological matrix was considered by using RevBayes’ dynamic likelihood approach (62). The molecular data were complete mitogenomes without the D-loop region obtained from ref. 31. We used the GTR+Γ+I model of nucleotide sequence evolution to model each molecular partition, which accounted for rate variation among sites, as well as for invariable loci.

We ran the phylogenetic inference analysis for eight million Markov chain Monte Carlo (MCMC) generations. We visually inspected that the run achieved convergence and good mixing using trace plots and that all parameters had an effective sample size >1,000 using the package “coda” v.0.19-4 (63) in R v.4.0.2 (64). After discarding 25% of the chain as burn-in, we randomly sampled 1,000 phylogenies (Dataset S3) from the posterior sample that were used in the subsequent analyses after removing the outgroup taxa (*Gorilla gorilla* and *Pan troglodytes*) and Denisovan (which has no cranial capacity estimate available). For all prior specifications and more detailed implementation, see *SI Appendix*.

### Cranial Capacity and Body Mass Estimates.

We assembled the largest collection, to our knowledge, of brain and body size estimates ever compiled for fossil hominins—ranging from ~7 Ma to end of the Pleistocene (Dataset S4). We collected specimen-level cranial capacity or endocranial volume (cm^3^) and body mass (kg) from a combination of recent compilations and meta-analyses (e.g., refs. 65 and 66 and primary sources. We preferred cranial estimates obtained directly from endocasts (either virtual or physical) over other methods (*SI Appendix*). For a few individuals, we used adult-projected values as available in the literature (n = 9) (*SI Appendix*).

All body mass estimates were obtained from the literature, e.g., refs. 65, 67, and 68 plus one additional estimate computed by us (*K. platyops,* KNM-WT 40000, Dataset S4). It is relevant to bear in mind that these body mass estimates are subject to error, as they are not directly measured values but mostly derived from regression models and as such subject to future change and revision. When available, body mass estimates calculated from lower limb anatomical elements (e.g., femur, tibia, etc.) or pelvic remains were generally preferred over those computed using upper limb, axial, and/or cranial remains), as it is largely agreed that weight-bearing skeletal elements correlate better with an individual’s body mass (67). Only body mass data from adult individuals were collected. Given the above, we then generated 1,000 datasets (Dataset S5) to account for different uncertainty sources in the following manner.

Age: Each specimen was assigned a randomly sampled age obtained from their specific temporal range based on the most updated dating information (*SI Appendix*) using a uniform distribution. Each specimen was assigned to a species based on consensus information (69), as well as on the hypodigm used by ref. 58 and in our phylogenetic inference analysis.

Taxonomy: For the specimens in which the taxonomic assignments were more controversial or unclear, we allowed them to be randomly classified as one of the species proposed for them in each of the 1,000 datasets. To give an example, if an individual has been classified as either *H. neanderthalensis* or *H. heidelbergensis,* we randomly allowed this specimen to be classified as *H. neanderthalensis* or *H. heidelbergensis* in different datasets.

The data compilation procedure described thus far (i.e., the collection of cranial capacity, body size, age, and taxonomy) resulted in a dataset comprising a total of 285 individuals. However, 184 specimens with cranial capacity did not have associated body mass estimates. For species where n < 20, we assigned each specimen a body size randomly sampled from within the observed range of estimates for that species. For species with larger sample sizes, we similarly performed random sampling, but explicitly considered geological age and corresponding rock unit (e.g., Chibanian, Calabrian, etc.) according to their sampled date, as well as the specific biogeographical realms (e.g., Afrotropical, Paleartic, Indomalayan, etc.) (70) based on their geographical location (Dataset S4). All these procedures were repeated a thousand times which resulted in a thousand hominin datasets (Dataset S5). Both body mass and cranial capacities were log_10_-transformed prior to the modeling step.

### Within- vs. between-Species Effects Using Bayesian PGLMM.

We applied Bayesian PGLMM (33, 71) to assess the relationship between cranial capacity, body mass, and time while also considering phylogenetic relatedness (*SI Appendix*). In PGLMM, the phylogenetic information is incorporated by adding a random effect that assumes that phylogenetic effects are correlated according to a phylogenetic variance–covariance (or correlation) matrix. In our case, we computed 1,000 matrices using our sample of phylogenies. To assess the relationships between cranial capacity, body mass, and time at both intra- and interspecific levels, we used a technique known as within-group centering (35) which separates each predictor variable into two components: one containing the group-level mean of each predictor (i.e., the species-level means of body mass and time) and a second one containing the within-group variability, (the difference between each specimen and the specific mean).

We accounted for possible slope differences per species by including species-specific random effects for the within-group variability of time (Model 1). We then repeated the modeling procedure incorporating species-specific random effects for the within-group variability of both time and body mass (Model 2). To assess the role of cladogenesis vs. anagenesis on encephalization, we carried out an additional modeling scenario (Model 3) by including the number of nodes between the root and each tip present in the phylogeny (log_10_ node count) into Model 1. Finally, to test whether there was an accelerating relative brain size increase through time we ran an additional set of models (Model 4) including an interaction term between- and within-species time as covariates. Values reported in the main text correspond to results obtained from Model 1 as no significant within-body mass effect was found in our models, while Table 1 shows the results obtained for Models 1 to 4.

Phylogenetic signal was measured using a modified version of Lynch’s heritability *h*^2^ that remains valid for variance–covariance matrices computed from nonultrametric trees (37) (*SI Appendix*). As suggested by ref. 38, we computed both marginal (variance explained by fixed effects) and conditional (variance explained by both fixed and random effects) R^2^ values as measures of goodness-of-fit, as these two measures are especially designed to deal with the most common problems faced when generalizing R^2^ for mixed-effects models. All the above-mentioned steps were implemented using the “MCMCglmm” v.2.33 (72) R package. We used a diffuse normal distribution centered around zero (μ = 0) with very large variance (σ^2^ = 10^8^) as prior for the fixed effects, while for the variances of the random effects, inverse-Gamma distributions with shape (α) and scale (β) parameters equal to 0.01 were applied. Burn-in time was 10,000 runs, and the total number of iterations was 1,000,000, with a thinning interval of 500.

Convergence and mixing were visually assessed by looking at the trace plots of each one of the fixed and random effects. All chains were run multiple times to ensure convergence, and we checked that effective sample sizes were >1,000. Every model tested was repeated 1,000 times using the previously mentioned datasets and phylogenies. By repeating our analyses in this way, using different phylogenies and datasets, we accounted for phylogenetic uncertainty as well as the uncertainty associated with chronometric ages and body mass data. We deemed an effect to be statistically significant when the pMCMC values obtained from the 1,000 analyses carried out for each one of our different modeling scenarios (*SI Appendix,* Table S2) were less than or equal to 0.05 in 95% of the cases. pMCMC is defined as twice the posterior probability that the estimate is either negative or positive, whichever probability is smaller (36). An R script is available to run Model 1 using the provided datasets and phylogenies (*SI Appendix*).

### Assessing Alternative Modeling Scenarios.

Our approach incorporates numerous sources of uncertainty as well as explicitly testing several hypotheses regarding hominin encephalization (Fig. 1). However, there will always be some level of controversy associated with taxonomic affiliation as well as the methods and approaches used to measure values like dates and traits. We therefore assessed several additional modeling scenarios, incorporating alternative data-treatments or species-assignments to determine the impacts of such uncertainty on our analyses.

To do this, we repeated the analyses described above but using several different modeling scenarios, which we will briefly list here. However, all models and their justifications are described in detail in the *SI Appendix*—and a complete list with associated numerical results can be found in *SI Appendix,* Tables S2–S5. Briefly, our additional analyses include models: incorporating alternative species classifications accounting for taxonomic uncertainty, removing all species with only a single cranial capacity estimate, combining *H. ergaster,* Georgian *H. erectus,* and *H. erectus* into a single species, combining *H. habilis* and *H. rudolfensis* into a single species, accounting for different methodological approaches to estimating cranial capacity, body size, and dating, assessing an alternative approach to filling in missing body sizes using phylogenetic imputation rather than random sampling, removing or replacing all cranial-estimated body sizes, excluding all young specimens for which adult-projected estimates were used, testing for alternative cranial capacity estimates for the *P. boisei* specimen KNM-WT 17400 (24), assessing whether the small-brained *H. naledi* and *H.* floresiensis behave differently from the rest of the sample, and finally, excluding *Sahelanthropus* owing to ongoing controversy regarding its hominin status (72, 73).

None of the additional modeling scenarios result in any substantial change to the results presented in the main text of this work. This not only shows the robustness of our results to numerous sources of uncertainty but also the flexibility of our approach to incorporate different hypothetical scenarios.

## Supplementary Material

Appendix 01 (PDF)

Dataset S01 (XLSX)

Dataset S02 (TXT)

Dataset S03 (TXT)

Dataset S04 (XLSX)

Dataset S05 (CSV)

## Data Availability

All study data are included in the article and/or supporting information.
