# Supplementary material for: Hominin brain size increase has emerged from within-species encephalization
Source: Proc Natl Acad Sci U S A. 2024 Nov 26;121(49):e2409542121. doi: 10.1073/pnas.2409542121 (PMC11626186; doi:10.1073/pnas.2409542121)
Supplement: Supplementary file 1 — Appendix 01 (PDF) [file pnas.2409542121.sapp.pdf]

PNAS

Supporting Information for

Hominin brain size increase has emerged from within-species encephalization

Thomas A. Püschel, Samuel L. Nicholson, Joanna Baker, Robert A. Barton, Chris Venditti

Thomas A. Püschel, Chris Venditti
Email: thomas.puschel@anthro.ox.ac.uk; c.d.venditti@reading.ac.uk

This PDF file includes:

Supporting text
Figure S1
Tables S1 to S5
Legends for Datasets S1 to S5
SI References

Other supporting materials for this manuscript include the following:

Datasets S1 to S5

Supporting Information Text

A brief introduction to phylogenetic linear mixed models

In our work we apply phylogenetic generalized linear mixed models (PGLMMs), which are methods that have been successfully developed and used before in other evolutionary biology publications, see e.g., (1–4), among others. However, we decided to provide a more detailed explanation about the PGLMMs we applied in our work below by providing a brief introduction to them.

Imagine we have phenotypic data, such as hominin cranial capacity, for several species, along with an associated covariate of interest—let's call it x (e.g., body mass). Additionally, we have access to a phylogeny from which we've derived a phylogenetic correlation matrix Σ, perhaps using a classical Brownian motion model, though alternative models could also apply. How can we construct a mixed model that accounts for the phylogenetic structure in order to explore the relationship between y and x?

A simple model can be outlined as follows:

$$y = \mu + \beta x + a + e$$

Where μ and β, are the intercept and slope for the covariate x, a is the phylogenetic random effect, and e is the residual error. The last two terms are assumed to be normally distributed with:

$$a \sim \mathcal{N}\ (0, \sigma_p^2 \Sigma)$$
$$e \sim \mathcal{N}\ (0, \sigma_R^2 \mathrm{I})$$

In this context, I represents the relevant identity matrix, whilst phylogenetic effects are correlated according to the phylogenetic correlation matrix Σ. In addition, our model estimates two variances: σ_p^2, which denotes the variance of the phylogenetic effect, and σ_R^2, which represents the residual error encompassing environmental effects, intraspecific variance, measurement error, and other potential factors.

Based on the above model, we can now consider a slightly more complex model that accounts for multiple measurements per species (e.g., multiple hominin specimens per species). Extending the model to handle this situation is straightforward, and we have:

$$y = \mu + \beta x + a + s + e$$

Where:

$$s \sim \mathcal{N}\ (0, \sigma_S^2 \mathrm{I})$$

Here, s represents the 'multiple measurement effect' or the species-specific effect once the phylogenetic influence has been taken out. This effect accounts for the variability that has been caused by the species' contingent characteristics (or species-specific effects); σ_S^2 is the variance of this effect. When considered together, $\sigma_p^2 \Sigma$ and $\sigma_S^2 \mathrm{I}$ account for the between-species variability (the first being caused by shared evolutionary history, while the second by contingent events). Please note that this second model assumes the same intraspecific variance for all the species in the dataset. Hence, in its current form, our second model does not distinguish between intra vs inter-specific effects (i.e., the type of relationship the slope β is measuring). If the covariate x only includes one value per species (i.e., mean specific values), we can rewrite our second model, considering an individual j belonging to species i, in the following manner:

$$y_{i,j} - a_i - s_i = \mu + \beta x_i + e_{i,j}$$

Thus, we can now consider the random effect a_i and s_i as within-species centering effects and the slope β as a between-species slope.

The situation is just slightly more complicated when using individual measurements in x. However, it is still possible to obtain the between-species and within-species slopes by using the within-group centering technique cited in our work (5, 6). The principle of this technique is to separate the predictor x into two components: one representing the group-level mean of x (i.e., the species-specific mean) and the other capturing the within-group variability (i.e., the differences among specimens relative to their specific mean). For an individual j belonging to species i, we can rewrite our previous model as follows:

$$y_{i,j} = \mu + \beta_B \overline{x}_i + \beta_w\left(x_{i,j} - \overline{x}_i\right) + a_i + s_i + e_{i,j}$$

Where:

$$\overline{x}_i = \frac{1}{J_i}\sum_{j=1}^{J_i} x_{i,j}$$

J_i corresponds to the number of individuals in species i. Therefore, in this model we are now fitting two slopes: β_B is the slope of regression between species, whereas $\beta_w$is the slope of regression within each species. Please note that, by definition, the between- and within- species predictors (i.e., $\overline{x}_i$ and $x_{i,j} - \overline{x}_i$) are orthogonal, and as such non-collinear.

All the models presented in our work are based on the above framework, but rather than having a single initial covariate (body mass), we also considered time (i.e., a total of four predictors, between-species body mass and time, and within-species body mass and time). Moreover, we made our models slightly more complicated as in some cases (see methods) by including one slope per species for the within species predictors (the so-called random slope models).

Phylogenetic signal

A standard phylogenetic generalized linear mixed model (PGLMM) is equivalent to Pagel's λ model of phylogenetic signal inference (2, 7), which means that if a phylogenetic correlation matrix is used it is possible to use Lynch's phylogenetic heritability h^2 as a measure of phylogenetic signal (4, 8, 9). Hence, we modified the calculation of h^2 to account for the extra random effects, as well as to consider the non-ultrametricity of our phylogenies by using a mathematical framework to estimate variance components using PGLMM that remains valid for variance-covariance matrices from non-ultrametric trees (10):

$$h^2 = \frac{\sigma_P^2\,\tau}{\sigma_P^2\,\tau + \sigma^2_{log_{10}bm_{within}|species} + \sigma^2_{time_{within}|species} + \sigma_R^2}$$

Where σ_P^2 is the estimated variance of the phylogenetic effect, τ is an arbitrary time, $\sigma^2_{log_{10}bm_{within}|species}$ is the variance of body mass within species variability, $\sigma^2_{time_{within}|species}$ is the temporal within species variability and σ_R^2 is the residual error variance. This was the calculation done for Model 2, whilst the computation for Model 1 is the same one but without the $\sigma^2_{log_{10}bm_{within}|species}$ term (this was also coherently adapted based on the different random effects used in the different models summarized in below and in Table S2). This means that to correctly estimate the heritability, σ_p^2 must be multiplied by an arbitrary time τ, which in our case corresponded to the median sampling time of all the tips. Hence, h^2 can be interpreted in this case as a phylogenetic signal estimate that applies to all the hominin sample, represented by a hypothetical individual that was sampled at time τ (i.e., the median sampling time of all the tips).

Extended phylogenetic results

We carried out a 'combined-evidence' Bayesian phylogenetic analysis as we needed reliable hominin phylogenies before running our phylogenetic comparative analyses. After discarding a 25% burn-in we obtained a posterior distribution of 60,000 phylogenetic trees from which we computed a maximum a posteriori (MAP) tree as a way of summarizing our posterior tree sample. Overall, the tree is well-resolved showing high posterior support with ~70% of the nodes displaying posterior values larger than 0.5 (Figure S1, Table S1). The part of the tree comprising *H. erectus*, Georgian *H. erectus, H.* ergaster, *H. naledi* and *H. floresiensis* was the most variable, showing the lowest posterior values. The topology of our MAP tree (Supp Fig. 1) differs from the recent trees obtained by the 'combined-evidence' analysis done by(11) mainly in the position of *H. naledi*, *H. floresiensis* and *Au. africanus.* Our divergence time estimates (Table S1) are in general agreement with the results obtained by the same study(11), as there is considerable overlap between the posterior density intervals (HPD) of both analyses.

Extended materials and methods

Phylogenetic analyses.

A 'combined-evidence' Bayesian phylogenetic analysis of extant and fossil hominin species, combining morphological and molecular data as well as stratigraphic range data from the fossil record e.g.,(12–14), was carried out to infer hominin phylogenetic relationships using RevBayes v.1.1.0 (15). The stratigraphic ranges are the first and last occurrences observed for a single species in the fossil record (Dataset S1, also see below). For all extant taxa, the minimum occurrence date was set to 0.0 Ma.

We used a 'Fossilized Birth Death Range Process' (FBDRP) (16) prior on the tree topology, which allows us to incorporate not only separate likelihood components associated with molecular and morphological data, but also these stratigraphic information as part of our tree inference. We used a log-normal prior to model both speciation λ (μ= -0.78, σ=0.68), and extinction μ rates (μ=-0.84, σ=0.8) based on estimates from ref(17). An extant sampling proportion (ρ) of 0.6 was used as not all extant Homininae species were sampled (*Pan paniscus* and *Gorilla beringei* were not included), whilst an exponential prior (ψ) of 10 was used to account for fossil sampling rate *Gorilla gorilla and Pan troglodytes* were treated as outgroup taxa and a uniform distribution between 8.0 and 12.5 Ma based on ref (18) was used as a prior on origin time (φ).

Our analyzed molecular and morphological datasets are the same ones used by refs (19, 20) with some modifications. The morphological data came from ref (21) and comprised a supermatrix of 391 craniodental characters. We removed *H. antecessor* from this matrix as it corresponds to a single juvenile individual with mostly missing data in the original dataset (21). We also added additional morphological characters that were originally coded as missing in two species (i.e., *Au. anamensis* and *H. floresiensis*) using information from refs (22, 23) (Dataset S2). The Mkv+Γ model (24) was used for the morphological data, which was partitioned into unordered and ordered characters, and then further partitioned based on the maximum number of character states of each division. Possible ascertainment bias in the morphological matrix was considered by using RevBayes' dynamic likelihood approach (25). The molecular data were complete mitogenomes without the D-loop region obtained from ref (19). We used the GTR+Γ+I model of nucleotide sequence evolution to model each one of these partitions, which accounted for rate variation among sites, as well as for invariable loci. An uncorrelated log-normal relaxed clock model with exponentially distributed hyperpriors (μ=2.0, σ^2=3.0) (26) was used for modelling branch rate variation among lineages for both the molecular and morphological datasets.

We performed the phylogenetic inference analysis using 8,000,000 Markov chain Monte Carlo (MCMC) generations. We visually inspected that the run achieved convergence and good mixing using trace plots, and that all parameters had an effective sample size >1000 using the package 'coda' v.0.19-4 (27) in R v.4.0.2 (28). After discarding a 25% burn-in we obtained a posterior distribution of 60,000 phylogenetic trees from which we computed a maximum a posteriori (MAP) tree as a way of summarizing our posterior tree sample (Fig. 2a). This MAP tree corresponds to

the tree topology that has the greatest posterior probability, averaged over all branch lengths and substitution parameter values, hence being an effective way of summarizing multiple phylogenies by determining which tree topology has been sampled the most often in the MCMC. This is the tree visualized in Figure 2. We randomly sampled 1,000 phylogenies (Dataset S3) from the posterior that were used in the subsequent analyses after removing the outgroup (*Gorilla gorilla* and *Pan troglodytes*) and Denisovan because this latter taxon does not have any cranial capacity estimate available.

Dates

We collated a database of hominin fossil specimens and their ages. Information of fossil ages was selected on the following criteria: 1) the most recent study of the fossil age; 2) dates obtained through direct methods applied to the fossil; and 3) dates obtained from the same stratigraphic layer/sediments as the fossil. Where neither criterion 2) or 3) could be met, we used 4) a date obtained from the archaeological site. One repeated issue of dating methods is that either minimum (the specimen is older than the age) or maximum (the specimen is younger than the age) ages are provided. In these cases, we applied a secondary set of criteria: 1) For direct fossil minimum ages or indirect stratigraphic/sediment minimum ages, the ages from the underlying stratigraphic layer were used to provide a maximum age boundary; 2) For direct fossil maximum ages or indirect stratigraphic/sediment maximum ages, the ages from the overlying stratigraphic layer were used to provide a maximum age boundary; 3) Where no suitable maximum/minimum age could be obtained from underlying/overlying stratigraphic sediments, we then default to the reported marine isotope stage (MIS) boundaries for an upper/lower age bracket. Together, these criteria allowed us to develop a comprehensive database with the most up-to-date information of the hominin fossil record.

Cranial capacity and body mass estimates

We assembled a database of cranial capacities and body masses for hominins ranging from ~ 7 Ma to end of the Pleistocene (i.e., 11.7 ka) (Dataset S4). To our knowledge, this is the largest collection of brain and body size estimates ever compiled for fossil hominins. Most of the specimen-specific data (i.e., cranial capacity or endocranial volume, in cm^3, and body mass, in kg) was obtained from recent studies and meta-analyses e.g.,(29, 30), but additional specimens were also collected from primary sources. In the case of cranial capacity, specific sources of these data were recorded, as well as the method used to compute these estimates (e.g., endocast, virtual endocast, regressions, etc.). In general, estimates obtained from endocasts (either physical or virtual) were preferred, followed by direct estimation methods (e.g., seeds or water), then by estimates obtained using regression equations, and finally by estimates obtained using any other methods. Most of the collected data corresponded to adult individuals, but we also included cranial capacities obtained from a few younger individuals. It is well-known that human absolute brain size increases rapidly during early development with a brain size at birth being around 27% of the adult size and reaching a 90% of adult brain size by the age of five (i.e., only a year later than the chimpanzee average) (31). As a result, for the few estimates available for individuals younger than seven years old at their death, we used their adult-projected values as available in the literature.

The data on hominin body mass estimates were obtained from the literature e.g. (29, 32, 33) plus one additional estimate computed by us (i.e., an estimate for *K. platyops* KNM-WT 40000, Supplementary dataset 4). It is relevant to bear in mind that that these body mass estimates are subject to error, as they are not directly measured values but mostly derived from regression models and as such subject to future change and revision. These body size estimates were collected at the specimen level and their provenance was recorded, as well as the anatomical element used to compute these estimates. When available, body mass estimates calculated from lower limb anatomical elements (e.g., femur, tibia, etc.) or pelvic remains were generally preferred over those computed using upper limb, axial and/or cranial remains), as it is largely agreed that weight-bearing skeletal elements correlate better with an individual's body mass (32). When multiple body mass estimates were available, those that met the above criteria and that were more recent were generally preferred. Only body mass data from adult individuals were collected.

We generated 1,000 datasets (Dataset S5) using this information to account for different uncertainty sources in the following manner. Each specimen was associated with an age range based on the most updated dating information available as described above, and then they were assigned a randomly sampled age obtained from their specific temporal range using a uniform distribution. This value was used as a time variable. The specimens were classified to the species level based on the consensus information available in the literature (34), as well as on the hypodigm used by ref (21), as the morphological dataset from this publication was used in our phylogenetic inference analysis. For the specimens in which the taxonomic assignments were more controversial or unclear, we allowed them to be randomly classified as one of the species proposed for them in each of the 1,000 datasets. To give an example, if an individual has been classified as either *H. neanderthalensis* or *H. heidelbergensis* we randomly allowed this specimen to be classified as *H. neanderthalensis* or *H. heidelbergensis* in different datasets. This process resulted in 285 specimens with estimated cranial capacities available.

However, only 101 of these specimens had both cranial capacities and body mass estimates available. For the remaining 184 specimens with only cranial capacity, body masses were sampled using the following criteria. For all the species with less than 20 specimens with cranial capacity available (i.e., *Au. afarensis, Au. africanus, Au. garhi, H. habilis, P. boisei, P. robustus,* and *H. ergaster*), we randomly sampled within the body mass estimates available for each one of these species (i.e., a species-specific sampling). In the case of *H. erectus* we applied the same procedure due to the reduced number of body mass estimates available for this species as compared to their total number of available cranial capacity estimates. A more complex body mass sampling procedure was applied for those species with more data available (i.e., *H. heidelbergensis, H. neanderthalensis* and *H. sapiens*). In these cases, each specimen was classified into a specific geological age and corresponding rock unit (e.g., Chibanian, Calabrian, etc.) according to their sampled date, as well as into specific biogeographical realms (e.g., Afrotropical, Paleartic, Indomalayan, etc.)(35) based on their geographical location. Using these two criteria, we sampled species-specific body mass estimates for each specimen using the available body masses for each subcategory if available (e.g., Chibanian-Afrotropical, Stage 4-Afrotropical, Stage 4-Indomalayan and so on) (Dataset S4). All these procedures were repeated a thousand times which resulted in the 1,000 hominin datasets. This sampling procedure does not 'break' the link between body mass and cranial capacity, as the average correlation value between these two traits for the 1,000 generated datasets is 0.63, which is almost exactly the same value as the one obtained when computing the correlation between cranial capacity and body mass using only the 101 individuals without imputed body mass values (i.e., 0.64). Both body mass and cranial capacities were $\log_{10}$-transformed prior to the modelling step.

Within- vs. between-species effects using Bayesian phylogenetic generalized linear mixed models

We applied Bayesian phylogenetic generalized linear mixed models (PGLMM) (4, 36) to assess the relationship between cranial capacity, body mass, and time considering phylogenetic relatedness (see above for further details about PGLMMs). This model is like standard PGLS but not only estimates the variance of the phylogenetic effect, it also incorporates a residual error term that can account for factors such as intraspecific variance, environmental effects, measurement error, among many others (8). In PGLMM, the phylogenetic information is incorporated by adding a phylogenetic random effect that is assumed to be normally distributed with a variance that assumes that phylogenetic effects are correlated according to a phylogenetic variance-covariance (or correlation) matrix. In our case, we computed these matrices using the 1,000 hominin phylogenies previously mentioned.

Since we were interested in assessing the relationships between cranial capacity, body mass and time both at the intra- and inter-specific levels, we applied a repeated measurements approach that allow us to obtain the between-species and within-species variables using a technique known as 'within-group centering' (6). The principle of this technique is to separate each predictor variable into two components: one containing the group-level mean of each predictor (i.e., the

species mean of each predictor; in our case body mass and time) and a second one containing the within-group variability, which is simply the difference between each specimen and their specific mean. In addition, we accounted for possible slope differences per species (i.e., a random slope model) by using a random effect (i.e., species-specific random effects for the within-group variability time; Model 1). We also repeated the above modelling procedure but incorporating another random effect (i.e., the same model but with species-specific random effects for the within-group variability time and body mass; Model 2). By using this modelling approach, we were thus able to estimate intraspecific variance and between-species slopes for multiple measurement data with the help of two additional random effects. To assess the role of cladogenesis vs. anagenesis on encephalization, we carried out an additional modelling scenario (Model 3) similar to Model 1 but including a new covariate (i.e., $\log_{10}$ node count) that can be considered as a speciation rate metric obtained by counting the number of nodes between the root and each tip present in the phylogeny. To test whether there was an accelerating relative brain size increase through time we ran an additional set of models (Model 4) including an interaction term between- and within-species time as covariate. Values reported in the main text correspond to results obtained from Model 1 as no significant within-body mass effect was found in our models, whilst Table 1 shows the results obtained for Models 1-4.

Phylogenetic signal was measured using a modified version of Lynch's heritability h^2 that remains valid for variance-covariance matrices computed from non-ultrametric trees (37). R^2 values were used as measures of goodness-of-fit of our models and were computed following the suggestions provided by ref (38), who recommended two R^2 metrics for mixed-effects models (i.e., marginal, and conditional R^2). These two metrics were especially designed to deal with the most common problems faced when generalizing R^2 for mixed-effects models. Marginal R^2 is concerned with variance explained by fixed effects, whilst conditional R^2 deals with variance explained by both fixed and random effects (38). All the above-mentioned steps were implemented using the 'MCMCglmm' v.2.33(73) R package. We used a diffuse normal distribution centered around zero (μ=0) with very large variance (σ^2=10^8) as prior for the fixed effects, whilst for the variances of the random effects, inverse-Gamma distributions with shape (α) and scale (β) parameters equal to 0.01 were applied. Burn-in time was 10,000 runs, and the total number of iterations was 1,000,000, with a thinning interval of 500. Convergence and mixing were visually assessed by looking at the trace plots of each one of the fixed and random effects. All chains were run multiple times to ensure convergence and we checked that effective sample sizes were > 1,000. Every model tested was repeated 1,000 times using the previously mentioned datasets and phylogenies. By repeating our analyses in this way, using different phylogenies and datasets, we accounted for phylogenetic uncertainty as well as the uncertainty associated with chronometric ages and body mass data. We deemed an effect to be statistically significant when the pMCMC values obtained from the 1,000 analyses carried out for each one our different modelling scenarios (see below and Table S2) were less than or equal to 0.05 in 95% of the cases. pMCMC is defined as twice the posterior probability that the estimate is either negative or positive, whichever probability is smaller (39). An R script is available to run Model 1 using the provided datasets and phylogenies (Supplementary information 5).

Assessing alternative modelling scenarios
To assess several additional modelling scenarios, we repeated the above procedures by running additional modelling sets (See Table S2 for a complete list). The numerical results of each model are reported in Tables S3-S5.

We first assessed a simpler model that did not include varying slopes and intercepts for the within-body mass and within-time effects. We ran this model to determine whether incorporating varying slopes would enhance the model's performance, which, in fact, they did. This model was assigned the model code AM1 in Tables S2-S5.

To assess the influence of including species with particularly small hypodigms, we carried out 1,000 analyses (Model 1) removing all the species that had a single cranial capacity in our

dataset (i.e., *S. tchandensis, Ar. ramidus, Au. anamensis, Au. garhi, K. platyops, Au. sediba,* and *H. floresiensis*). This model was assigned the model code AM2 in Tables S2-S5.

We assessed if consolidating *H. ergaster*, Georgian *H. erectus* and *H. erectus* into a single species could influence the observed pattern by running 1,000 analyses (Model 1) using the '*H. erectus* sensu lato' category for these three species. This model was assigned the model code AM3 in Tables S2-S5.

We ran 2,000 analyses (i.e., 1,000 each for Models 1 and 2, respectively) using an alternative dataset that considered a different cranial capacity value for the P. boisei specimen KNM-WT 17400 (500 cm^3 rather than 400 cm^3) as these two highly dissimilar estimates have been used by different sources (40). These models are assigned the model code AM4 and AM5 in Tables S2-S5.

We ran 1,000 analyses (Model 1) using an alternative species classification that incorporated additional taxonomic uncertainty) to assess if different taxonomic categorizations could influence our results. 32 individuals were classified differently as compared to our original datasets (this alternative taxonomic classification can be found as column in Supplementary dataset 4). This model is assigned the model code AM6 in Tables S2-S5.

To assess the potential impact of different dating methodologies we ran additional models including the dating methodologies used to obtain the minimum and maximum age brackets for each specific individual as additional random effects. These models are assigned the model codes AM7-AM9 in Tables S2-S5.

We assessed the importance of disentangling intra- and inter-specific levels of variation when analyzing brain size evolution by running two additional sets of models – one that did not account for the differences, and another using a PGLMM formulation that is equivalent to a standard phylogenetic generalized least squares (PGLS) regression that uses overall species means for body size and time. These models are assigned the model codes AM10 and AM11 in Tables S2-S5.

We assess the potential anagenetic pattern of brain size evolution by running an additional model excluding the within- and between-time effects whilst still estimating node count. This model is assigned the model code AM12 in Tables S2-S5.

To assess the potential impact of our random-sampling imputation procedure described in the 'Cranial capacity and body mass estimates' section, we also tested a phylogenetic imputation procedure to estimate missing body mass values in our hominin sample. In the same way as described before, we started by associating each specimen with an age range based on the most updated dating information available to then assign them a randomly sampled age obtained from their specific temporal range. Then, the specimens were classified to the species level, and for those specimens in which the taxonomic assignments were unclear, we allowed them to be randomly labelled as any of one of their proposed species in different datasets as was also done previously. This resulted in 1,000 datasets, with every specimen having an associated date obtained from their temporal range, and a taxonomic label that may differ between different datasets depending on how uncertain or not were the available taxonomic classifications available in the literature(34). We then took these 1,000 datasets and 'paired' them with our 1,000 phylogenies previously described. We then applied a phylogenetic imputation procedure using PhyloPars (41)(42) to impute our missing data. This procedure corresponds to a statistical framework that allowed us to estimate phylogenetic trait covariance while accounting for both within-species variation and missing data. Since we had multiple within-species observations, PhyloPars allowed us to estimate both within-species (i.e., phenotypic) trait covariance, as well as among-species (i.e., phylogenetic) covariance. As in ref (3), phenotypic covariance was assumed to be equivalent among species, and we also assumed Brownian motion in our imputation procedure. Missing observations were incorporated by maximizing the log-likelihood of the

covariance parameters using all available data (41), thus allowing us to predict means and covariances for missing values at the tips of the phylogenetic tree. In our case, all available data consisted of three variables (i.e., cranial capacity, body mass and time), and we had three types of specimens in our dataset (i.e., individuals with the three variables present; individuals with cranial capacity and time available; and individuals with body mass and time available). Therefore, we phylogenetically imputed body mass values or cranial capacities depending on the specific individual, whilst considering both within-species trait covariance, as well as phylogenetic covariance among the three available variables (i.e., cranial capacity, body mass and time). This phylogenetic imputation procedure was carried out using the R package 'Rphylopars' v.0.3.9 (42). To make our results more comparable with our initial imputation procedure, we only retained the 285 specimens with original cranial capacities available for further analysis. This resulted in 1,000 datasets that were then used alongside the 1,000 phylogenies to run 1,000 'Model 2' (see above) to compare our results. This modelling procedure shows that, irrespective of the applied imputation procedure, our results are qualitatively equivalent with those obtained by our models analyzing the datasets generated using our initial sampling procedure, as we found again a significant within-species time and between-species body mass effects, with no significant results for the other variables (See Supplementary Table 2). This model was assigned the model code AM13 in Tables S2-S5.

As there is ongoing controversy regarding the hominin status of *S.tchadensis,* we also ran Model 1 excluding this species from our analyses. This model is assigned the model code AM 14 in Tables S2-S5.

We tested the potential impact of including specimens with adult-projected cranial capacities, by running Model 1 without them (i.e., we excluded AL 333-105, Gibraltar 2, Mojokerto, OH 13, OH 16, Shungura E-338Y-6, SK 27, MLD 3, and Taung 1). This model is assigned the model code AM15 in Tables S2-S5.

To evaluate the potential impact of how cranial capacities were estimated, we run Model 1 again but this time we included an additional random effect to our multilevel model to account for cranial capacity estimation methods, using the following categories: virtual endocast, endocast, regression, direct measurement (e.g., seeds/water displacement), and comparison. This model is assigned the model code AM16 in Tables S2-S5.

We evaluated whether combining *H. habilis* and *H. rudolfensis* into a single species could influence the observed pattern by running 1,000 analyses (Model 1) using the *H. habilis* sensu lato category for these two species. This model is assigned the model code AM17 in Tables S2-S5.

To assess if *H. naledi* and *H. floresiensis* behave differently from the rest of our sample, we ran Model 1 again, but in this case added a 'dummy code' variable distinguishing those individuals belonging to *H. naledi* and *H. floresiensis* from all the rest. The obtained results show that this 'dummy code' variable is not significant, which means that, after accounting for the effects of the continuous variables in our model, the dummy variable (i.e., being *H. naledi* or *H. floresiensis* vs. being any other hominin) does not explain a substantial amount of additional variance in the outcome variable. The fact that we found a non-significant effect for the dummy variable suggests that the difference between the two categories is not statistically meaningful in explaining variation in cranial capacity. In other words, once we control for the continuous variables in our model, being in one category versus the other does not lead to a significant difference in the cranial capacity. This model was assigned the model code AM18 in Tables S2-S5.

We assessed the potential role of body mass estimation methods in our analyses by allocating a new body mass estimate obtained from other skeletal elements to all the specimens that initially had body mass estimates obtained from cranial measurements by using the sampling procedure described in the 'Cranial capacity and body mass estimates' section. There were two species

(i.e., *S. tchadensis* and *K. platyops*) that did not have any non-cranial body mass estimates available, therefore they were removed from the 1,000 datasets and phylogenies. Then we ran Model 1 as previously described. Our results were again qualitatively identical. To further assess this, we also ran an extreme version of the above test in which we simply removed all the specimens that had body mass estimates obtained from cranial remains (i.e., we did not include their body mass or cranial capacity values). Then we ran Model 1 as described in the main text, and the results were again qualitatively identical the those originally reported. These models were assigned the model codes AM19 and AM20 in Tables S2-S5.

None of the above-mentioned additional modelling results changed the results presented in the main text of this work in any substantial way. In all cases, we find qualitatively identical conclusions. This not only shows the robustness of our results, but also the flexibility of our approach to incorporate different hypothetical scenarios.

Example R code to run Model 1 on a single dataset and phylogeny

```
# Load necessary packages
library(ape)
library(MCMCglmm)

# Read 1,000 phylogenies
phylos <-
read.tree("Supp_data3_1K_hominin_phylogenies_new.trees")
# Select a specific phylogeny, e.g., the 123rd one
phylo <- phylos[[123]]
# Create the phylogenetic variance-covariance matrix
inv.phylo <- inverseA(phylo, nodes = "all", scale = FALSE)

# Read dataset
datasets <- read.csv("Supp_data5_1K_datasets_new.csv")
# Select the specific dataset, e.g., dataset 123
data <- datasets[datasets$datasetnumber == 123, ]

# Prepare data
data$CC <- log10(data$Cranial.capacity)  # Log-transform
cranial capacity
data$BM <- log10(data$Body.mass)         # Log-transform
body mass
data$phylo <- data$Species               # Duplicate
species column for phylogeny

# Compute species-specific mean of body mass (BM)
data$spec_mean_bm <- sapply(split(data$BM, data$Species),
mean)[data$Species]
# Apply within-group centering for body mass
data$within_spec_bm <- data$BM - data$spec_mean_bm

# Compute species-specific mean of age
```

```
data$spec_mean_age <- sapply(split(data$Age, data$phylo),
mean)[data$phylo]
# Apply within-group centering for age
data$within_spec_age <- data$Age - data$spec_mean_age

# Set priors
prior <- list(
  G = list(
    G1 = list(V = 1, nu = 0.02),
    G2 = list(V = 1, nu = 0.02)
  ),
  R = list(V = 1, nu = 0.02)
)

# Model 1
model1 <- MCMCglmm(
  CC ~ spec_mean_bm + within_spec_bm + spec_mean_age +
within_spec_age,
  random = ~phylo + Species:within_spec_age,
  family = "gaussian",
  ginverse = list(phylo = inv.phylo$Ainv),
  prior = prior,
  data = data,
  nitt = 1000000,
  thin = 500,
  burnin = 10000,
  verbose = TRUE
)

# Display model results
# Note: Different results will be obtained with different
datasets and phylogenies.
# The results reported in the main text are the average
across 1,000 datasets and phylogenies.
summary(model1)

# Plot trace plots
plot(model1)
```

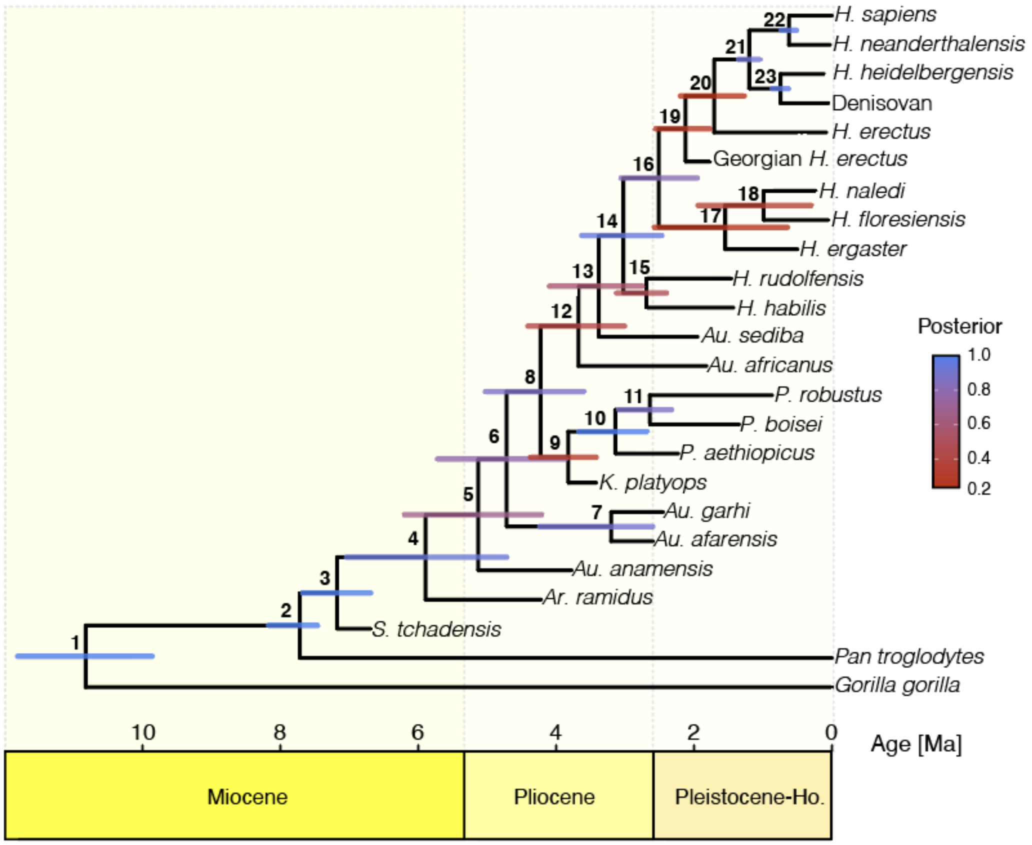

Fig. S1. Maximum a posteriori (MAP) tree summarising the hominin phylogenies obtained from a 'combined-evidence' Bayesian phylogenetic analysis. The length of the bars on the MAP tree correspond to the age 95% highest posterior density interval (HPD), while the colour represents posterior support. Numbers on the phylogeny correspond to node numbers in Supplementary table 1.

Table S1. Divergence time estimates and posterior support for the phylogenetic analysis.

Node number	Minimum bound for the Age 95% highest posterior density interval (HPD) [Ma]	Maximum bounds for the Age 95% highest posterior density interval (HPD) [Ma]	Mean divergence time [Ma]	Posterior
**1**	9.86	11.81	10.84	1
**2**	7.46	8.17	7.82	1
**3**	6.69	7.68	7.18	1
**4**	4.72	7.04	5.88	0.94
**5**	4.21	6.20	5.21	0.69
**6**	3.86	5.72	4.79	0.78
**7**	2.60	4.24	3.42	0.9
**8**	3.60	5.03	4.31	0.87
**9**	3.42	4.37	3.90	0.32
**10**	2.68	3.67	3.18	0.99
**11**	2.33	3.09	2.71	0.88
**12**	3.01	4.41	3.71	0.42
**13**	2.75	4.10	3.42	0.58
**14**	2.47	3.63	3.05	0.95
**15**	2.40	3.12	2.76	0.48
**16**	1.95	3.06	2.51	0.82
**17**	0.64	2.58	1.61	0.26
**18**	0.30	1.94	1.12	0.36
**19**	1.77	2.56	2.17	0.3
**20**	1.27	2.20	1.73	0.23
**21**	1.04	1.36	1.20	0.91
**22**	0.51	0.74	0.62	0.97
**23**	0.63	0.87	0.75	0.97

Table S2. List of all models that were carried out, including main models and alternative modelling scenarios.

Model	Model Code	Fixed effects	Random effects	Notes & Justification
Main models				
**Model 1**	M1	between-species body mass + within-species body mass+ between-species time + within-species time	phylogeny + species:within-species time	Model with varying slopes and intercepts for within-time effects
**Model 2**	M2	between-species body mass + within-species body mass+ between-species time + within-species	phylogeny + species:within-species body mass +species:within-species time	Model with varying slopes and intercepts for within-body mass and within-time effects
**Model 3**	M3	between-species body mass + within-species body mass+ between-species time + within-species time + node count	phylogeny + species:within-species time	Model used to assess cladogenesis vs. antimenesis
**Model 4**	M4	between-species body mass + within-species body mass+ between-species time + within-species + between-species time:within-species	phylogeny	Model used to test for accelerating evolution
Additional models				
**Simpler model**	AM1	between-species body mass + within-species body mass+ between-species time + within-species time	phylogeny	Model without varying slopes and intercepts for within-body mass and within-time effects
**Small hypodigm**	AM2	between-species body mass + within-species body mass+ between-species time + within-species time	phylogeny + species:within-species time	All species with a single specimen with cranial capacity were removed from the datasets and phylogenies

Model	Model Code	Fixed effects	Random effects	Notes & Justification
**Homo erectus sensu lato**	AM3	between-species body mass + within-species body mass+ between-species time + within-species time	phylogeny + species:within-species time	H. ergaster, Georgian H. erectus and H. erectus were condensed into a single category 'H. erectus sensu lato' in both the datasets and phylogenies
**Alternative cranial capacity for KNM-WT 17400 1**	AM4	between-species body mass + within-species body mass+ between-species time + within-species time	phylogeny + species:within-species time	Model 1 using alternative datasets that considered a different cranial capacity value for the P. boisei specimen KNM-WT 17400 (500 cm3 rather than 400 cm3)
**Alternative cranial capacity for KNM-WT 17400 2**	AM5	between-species body mass + within-species body mass+ between-species time + within-species	phylogeny + species:within-species body mass +species:within-species time	Model 2 using alternative datasets that considered a different cranial capacity value for the P. boisei specimen KNM-WT 17400 (500 cm3 rather than 400 cm3)
**Alternative taxonomic classification**	AM6	between-species body mass + within-species body mass+ between-species time + within-species time	phylogeny + species:within-species time	Model using datasets in which additional individuals were allowed to be classified into different species in different datasets
**Dating method 1**	AM7	between-species body mass + within-species body mass+ between-species time + within-species time	phylogeny +dating method maximum date +dating method minimun date	Model including dating methods for both the minimum and maximum dates as random effects
**Dating method 2**	AM8	between-species body mass + within-species body mass+ between-species time + within-species time	phylogeny +dating method maximum date +dating method minimu date + species:within time	Model including dating methods for both the minimum and maximum dates as random effects, as well as varing slopes for the within-species time effect
**Dating method 3**	AM9	between-species body mass + within-species body mass+ between-species time + within-species time	phylogeny +dating method maximum date +dating method minimu date+ species:within body mass + species:within time	Model including dating methods for both the minimum and maximum dates as random effects, as well as varing slopes for the within-species time and body mass effects
**Between-species only**	AM10	between-species body mass + between-species time	phylogeny	Model exclusively using the between-species variables for body mass and time as fixed effects

Model	Model Code	Fixed effects	Random effects	Notes & Justification
**Species means**	AM11	species-mean body mass + species-mean time	phylogeny	Model using species mean values for body mass and time (equivalent to a PGLS)
**Model node count and no time variables**	AM12	between-species body mass + within-species body mass+ node count	phylogeny + species:within-species time	Model used to assess the role of node count without the time-related variables
**Model 2 using phylogenetically imputed body mass values**	AM13	between-species body mass + within-species body mass+ between-species time + within-species	phylogeny + species:within-species body mass +species:within-species time	Model 2 using alternative datasets in which body mass values were phylogenetically imputed
**No S. tchadensis**	AM14	between-species body mass + within-species body mass+ between-species time + within-species time	phylogeny + species:within-species time	S. tchadensis was removed from the dataset and phylogeny
**No individuals with adult-projected cranial capacity values**	AM15	between-species body mass + within-species body mass+ between-species time + within-species time	phylogeny + species:within-species time	Individuals with adult-project cranial capacities were removed
**Cranial capacity estimation method**	AM16	between-species body mass + within-species body mass+ between-species time + within-species time	phylogeny + species:within-species + cranial capacity estimation method	An additional random effect was added to Model 1 to account for cranial capacity estimation methods
**Homo habilis sensu lato**	AM17	between-species body mass + within-species body mass+ between-species time + within-species time	phylogeny + species:within-species time	H. habilis and H. rudolfensis were condensed into a single category 'H.habilis sensu lato' in both the datasets and phylogenies
**Dummy-coded' Homo floresiensis and Homo naledi**	AM18	between-species body mass + within-species body mass+ between-species time + within-species + dummy code	phylogeny + species:within-species time	H. floresiensis and H. naledi were 'dummy-coded' and this predictor was added as fixed effect to assess if these two species behave differently from the rest of the sample
**Body mass estimates derived**	AM19	between-species body mass + within-species body mass+	phylogeny + species:within-species time	Body masses from individuals that originally had body mass estimates derive from cranial

Model	Model Code	Fixed effects	Random effects	Notes & Justification
**from cranial measurements removed**		between-species time + within-species + dummy code		measurements were replaced using data as described in the methods section. S. tchadensis and K. platyops had to be removed from this analysis due to the lack fo alternative body mass estimates.
**No individuals with body mass estimates from cranial measurements**	AM20	between-species body mass + within-species body mass+ between-species time + within-species + dummy code	phylogeny + species:within-species time	All individuals with body masses derived from cranial measurements were removed from the analysis

Table S3: Model fits for all tested models, including DIC, marginal and conditional R^2 value, and heritability (h^2).

Model Code	DIC	$R^2_{marg.}$ (mean)	$R^2_{marg.}$ (mode)	$R^2_{marg.}$ (L95CI)	$R^2_{marg.}$ (U95CI)	$R^2_{cond.}$ (mean)	$R^2_{cond.}$ (mode)	$R^2_{cond.}$ (L95CI)	$R^2_{cond.}$ (U95CI)	h^2 (mean)	h^2 (mode)	h^2 (L95CI)	h^2 (U95CI)
**M1**	-938.718	0.612	0.693	0.314	0.835	0.935	0.946	0.886	0.975	0.952	0.961	0.913	0.985
**M2**	-953.615	0.610	0.688	0.319	0.830	0.947	0.955	0.907	0.979	0.888	0.903	0.808	0.958
**M3**	-938.943	0.641	0.716	0.362	0.848	0.939	0.949	0.892	0.977	0.951	0.960	0.909	0.985
**M4**	-888.397	0.615	0.693	0.319	0.837	0.882	0.898	0.804	0.948	0.920	0.930	0.863	0.970
**AM1**	-882.182	0.613	0.693	0.315	0.837	0.879	0.896	0.798	0.947	0.918	0.928	0.859	0.969
**AM2**	-917.912	0.702	0.786	0.419	0.889	0.948	0.959	0.903	0.983	0.919	0.935	0.847	0.980
**AM3**	-926.911	0.569	0.659	0.239	0.820	0.931	0.943	0.878	0.975	0.955	0.964	0.917	0.987
**AM4**	-942.564	0.617	0.697	0.324	0.838	0.936	0.947	0.888	0.976	0.952	0.961	0.913	0.985
**AM5**	-957.064	0.616	0.693	0.328	0.833	0.947	0.956	0.909	0.979	0.888	0.902	0.808	0.958
**AM6**	-938.722	0.612	0.692	0.315	0.835	0.935	0.946	0.886	0.975	0.952	0.961	0.913	0.985
**AM7**	-869.290	0.485	0.537	0.209	0.726	0.917	0.925	0.870	0.957	0.882	0.922	0.750	0.977
**AM8**	-931.956	0.486	0.535	0.211	0.725	0.955	0.960	0.925	0.980	0.881	0.920	0.746	0.977
**AM9**	-947.520	0.487	0.537	0.215	0.724	0.962	0.967	0.938	0.983	0.878	0.920	0.740	0.977
**AM10**	-859.025	0.607	0.690	0.303	0.834	0.869	0.887	0.781	0.942	0.910	0.922	0.846	0.966
**AM11**	-40.223	0.601	0.690	0.272	0.852	0.783	0.843	0.565	0.949	0.799	0.868	0.578	0.965
**AM12**	-919.242	0.629	0.700	0.359	0.834	0.932	0.943	0.882	0.974	0.945	0.955	0.900	0.983
**AM13**	-935.157	0.663	0.732	0.411	0.849	0.947	0.955	0.909	0.978	0.949	0.961	0.902	0.986
**AM14**	-935.760	0.604	0.690	0.287	0.839	0.936	0.947	0.886	0.977	0.944	0.954	0.896	0.983
**AM15**	-915.987	0.592	0.670	0.293	0.822	0.934	0.944	0.885	0.974	0.955	0.963	0.917	0.986
**AM16**	-941.228	0.449	0.506	0.135	0.723	0.953	0.960	0.916	0.985	0.954	0.962	0.915	0.986
**AM17**	-933.622	0.599	0.684	0.288	0.831	0.934	0.945	0.884	0.976	0.953	0.962	0.912	0.986
**AM18**	-939.382	0.639	0.710	0.370	0.841	0.930	0.941	0.880	0.972	0.941	0.952	0.892	0.982
**AM19**	-930.222	0.5253	0.5928	0.1825	0.7921	0.9360	0.9484	0.8832	0.9794	0.954	0.9633	0.9151	0.9868
**AM20**	-767.234	0.6529	0.7419	0.3478	0.8673	0.9414	0.9533	0.8920	0.9806	0.939	0.9508	0.8862	0.9827

Numerical values correspond to the grand means of 1,000 models obtained for each scenario tested.

Table S4: Fixed effect parameter estimates and significance for all tested models.

Part 1 of 4.

Model Code	α	α (L95CI)	α (U95CI)	α (pMCMC)	α (Pct < 0.05)	BM_b	BM_b (L95CI)	BM_b (U95CI)	BM_b (pMCMC)	BM_b (Pct < 0.05)	BM_w	BM_w (L95CI)	BM_w (U95CI)	BM_w (pMCMC)	BM_w (Pct < 0.05)
**M1**	1.485	0.660	2.314	0.004	99.5%	0.792	0.341	1.238	0.005	99.4%	0.057	-0.015	0.128	0.210	29%
**M2**	1.467	0.640	2.304	0.004	99.7%	0.807	0.350	1.256	0.005	99.1%	0.063	-0.025	0.152	0.234	17.6%
**M3**	1.129	0.113	2.150	0.067	65.9%	0.833	0.375	1.285	0.004	99.3%	0.057	-0.015	0.128	0.210	28.4%
**M4**	1.490	0.674	2.313	0.004	99.2%	0.787	0.340	1.230	0.006	98.9%	0.061	-0.006	0.128	0.158	38.9%
**AM1**	1.489	0.670	2.317	0.004	99.4%	0.788	0.339	1.232	0.006	99.2%	0.057	-0.011	0.125	0.192	32.5%
**AM2**	1.156	-0.052	2.352	0.092	46.3%	1.038	0.372	1.710	0.011	97.8%	0.057	-0.015	0.128	0.209	28.2%
**AM3**	1.565	0.617	2.523	0.007	97.8%	0.741	0.216	1.258	0.020	90.0%	0.068	-0.003	0.140	0.134	43.0%
**AM4**	1.479	0.658	2.303	0.003	99.4%	0.797	0.348	1.240	0.005	99.4%	0.057	-0.014	0.128	0.206	28.9%
**AM5**	1.462	0.639	2.293	0.003	99.6%	0.811	0.358	1.259	0.005	99.5%	0.063	-0.024	0.152	0.230	18.0%
**AM6**	1.484	0.663	2.315	0.003	99.5%	0.793	0.342	1.240	0.006	99.4%	0.057	-0.015	0.128	0.210	28.3%
**AM7**	1.469	0.642	2.306	0.004	99.5%	0.804	0.349	1.253	0.005	99.4%	0.056	-0.013	0.127	0.211	30.9%
**AM8**	1.464	0.638	2.303	0.004	99.6%	0.808	0.352	1.259	0.005	99.3%	0.056	-0.018	0.129	0.229	26.6%
**AM9**	1.438	0.600	2.285	0.004	99.3%	0.826	0.363	1.283	0.005	99.6%	0.063	-0.027	0.154	0.245	16.6%
**AM10**	1.485	0.663	2.312	0.003	99.3%	0.792	0.341	1.237	0.005	99.2%					
**AM11**	1.471	0.460	2.486	0.009	99.5%										
**AM12**	1.062	0.302	1.831	0.019	91.7%	0.837	0.398	1.271	0.003	99.8%	0.058	-0.016	0.131	0.224	28.9%
**AM13**	1.133	0.254	2.020	0.019	95.8%	1.004	0.513	1.489	0.002	100.0%	0.124	-0.014	0.262	0.080	3.9%
**AM14**	1.515	0.626	2.409	0.006	98.6%	0.774	0.278	1.265	0.014	93.7%	0.057	-0.015	0.128	0.210	27.5%
**AM15**	1.505	0.676	2.340	0.003	99.5%	0.776	0.326	1.224	0.006	98.9%	0.061	-0.011	0.133	0.186	31.8%
**AM16**	1.482	0.647	2.321	0.004	99.4%	0.786	0.332	1.235	0.006	99.2%	0.059	-0.012	0.130	0.195	30.60%
**AM17**	1.493	0.618	2.377	0.005	99.8%	0.787	0.308	1.263	0.009	97.7%	0.068	-0.004	0.139	0.135	41.9%
**AM18**	2.053	1.137	2.974	0.001	100.0%	0.518	0.033	1.001	0.052	58.2%	0.057	-0.015	0.128	0.210	28.0%
**AM19**	1.900	1.020	2.781	0.006	100.0%	0.560	0.077	1.044	0.002	98.2%	0.031	-0.045	0.107	0.431	7.5%
**AM20**	1.488	0.590	2.388	0.008	97.1%	0.814	0.305	1.319	0.012	94.8%	0.041	-0.051	0.132	0.419	9.9%

BM_b = between-species body mass; BM_w = within-species body mass; t_b = between-species time; t_w = within-species time, NC = root-to-tip node count; t_b:t_w = interaction term between both within-species time and between-species time; BM_{sp} = mean species-level body mass; t_{sp} = mean species-level time; Hnal+Hfl = dummy code distinguishing *H. naledi* and *H. floresiensis* from other hominins. Numerical values correspond to the grand means of 1,000 modelling results obtained for each one of the modelling scenarios used, excepting when percentages are reported. Pct < 0.05 refers to the percentage of pMCMC values identified to be less than or equal to 0.05 from across the 1,000 analyses.

Table S4, cont.: Fixed effect parameter estimates and significance for all tested models.

Part 2 of 4.

Model Code	t_b	t_b (L95CI)	t_b (U95CI)	t_b (pMCMC)	t_b (Pct < 0.05)	t_w	t_w (L95CI)	t_w (U95CI)	t_w (pMCMC)	t_w (Pct < 0.05)
**M1**	-4.60023E-05	-9.22947E-05	7.06185E-07	0.069281818	42.2%	-6.59652E-05	-9.46513E-05	-3.74196E-05	0.000533333	100.0%
**M2**	-4.73056E-05	-9.36387E-05	-5.37637E-07	0.062254545	47.1%	-6.48863E-05	-9.4568E-05	-3.5115E-05	0.000634343	100.0%
**M3**	-8.28987E-06	-8.78428E-05	7.18085E-05	0.655487778	7%	-6.60028E-05	-9.46019E-05	-3.73826E-05	0.000570556	100.0%
**M4**	-0.0000455	-0.0000913	0.000000866	0.072187879	42.3%	-0.000110777	-0.00015261	-0.0000691	0.001010101	100.0%
**AM1**	-4.55969E-05	-9.1792E-05	9.02039E-07	0.070893333	41.9%	-6.60413E-05	-9.33273E-05	-3.87238E-05	0.000571111	100.0%
**AM2**	-5.46902E-05	-0.000142708	3.40132E-05	0.230765657	4.6%	-6.59914E-05	-9.45937E-05	-3.73567E-05	0.000540909	100.0%
**AM3**	-4.47959E-05	-9.56234E-05	7.00961E-06	0.100873737	17.3%	-9.67867E-05	-0.000119173	-7.42986E-05	0.000505051	100.0%
**AM4**	-4.63413E-05	-9.22785E-05	2.97806E-10	0.066020202	44.3%	-6.68294E-05	-9.52327E-05	-3.83158E-05	0.000525253	100.0%
**AM5**	-4.76338E-05	-9.36264E-05	-1.16394E-06	0.058993939	51.3%	-6.56865E-05	-9.51928E-05	-3.60894E-05	0.000576263	100.0%
**AM6**	-4.601E-05	-9.22043E-05	6.79112E-07	0.068725253	42.5%	-6.59825E-05	-9.46595E-05	-3.74159E-05	0.000533333	100.0%
**AM7**	-0.0000498	-0.0000965	-0.00000271	0.052295	57.2%	-0.0000575	-0.0000949	-0.0000201	0.00852278	97.1%
**AM8**	-0.0000503	-0.0000968	-0.00000294	0.050261667	61.2%	-0.0000575	-0.0000968	-0.0000183	0.011181667	95.8%
**AM9**	-0.0000511	-0.0000981	-0.0000035	0.048701111	61.7%	-0.0000572	-0.0000981	-0.0000162	0.017594444	91.2%
**AM10**	-4.59769E-05	-9.20392E-05	6.97233E-07	0.068723232	43.6%					
**AM11**										
**AM12**										
**AM13**	-4.74E-05	-9.13E-05	-7.19E-06	0.05	61.7%	-6.69E-05	-9.57E-05	-3.84167E-05	0.000526263	100.0%
**AM14**	-4.93E-05	-1.13E-04	1.55E-05	1.67E-01	16.5%	-6.60E-05	-9.46E-05	-3.74E-05	0.000581111	100.0%
**AM15**	-4.46E-05	-9.11E-05	2.40E-06	7.89E-02	36.2%	-8.41E-05	-0.000115716	-5.24E-05	0.000558333	100.0%
**AM16**	-4.45E-05	-9.09E-05	2.40E-06	8.01E-02	35.7%	-6.05E-05	-8.95E-05	-3.15E-05	0.000677222	100.00%
**AM17**	-4.61E-05	-9.36E-05	2.08E-06	7.56E-02	38.9%	-6.32E-05	-9.20E-05	-3.44E-05	0.000615556	100.0%
**AM18**	-6.31E-05	-1.07E-04	-1.81E-05	1.18E-02	99.8%	-6.60E-05	-9.46E-05	-3.73E-05	0.000580556	100.0%
**AM19**	-6.22E-05	-1.38E-04	1.40E-05	1.40E-01	24.8%	-6.62E-05	-9.49E-05	-3.76E-05	0.00053641	100.0%
**AM20**	-6.33E-05	-1.31E-04	4.52E-06	9.26E-02	38.7%	-6.59E-05	-9.82E-05	-3.35E-05	0.000728489	100.0%

BM_b = between-species body mass; BM_w = within-species body mass; t_b = between-species time; t_w = within-species time, NC = root-to-tip node count; t_b:t_w = interaction term between both within-species time and between-species time; BM_{sp} = mean species-level body mass; t_{sp} = mean species-level time; Hnal+Hfl = dummy code distinguishing *H. naledi* and *H. floresiensis* from other hominins. Numerical values correspond to the grand means of 1,000 modelling results obtained for each one of the modelling scenarios used, excepting when percentages are reported.

Table S4, cont.: Fixed effect parameter estimates and significance for all tested models.

Part 3 of 4.

Model Code	NC	NC (L95CI)	NC (U95CI)	NC (pMCMC)	NC (Pct < 0.05)	$t_b : t_w$	$t_b : t_w$ (L95CI)	$t_b : t_w$ (U95CI)	$t_b : t_w$ (pMCMC)	$t_b : t_w$ (Pct < 0.05)
**M1**										
**M2**										
**M3**	0.323	-0.231	0.881	0.287	5.9%					
**M4**						0.000000039	1.11E-08	6.69E-08	0.013469697	95.00%
**AM1**										
**AM2**										
**AM3**										
**AM4**										
**AM5**										
**AM6**										
**AM7**										
**AM8**										
**AM9**										
**AM10**										
**AM11**										
**AM12**	0.370	0.056	0.683	0.031	85.2%					
**AM13**										
**AM14**										
**AM15**										
**AM16**										
**AM17**										
**AM18**										
**AM19**										
**AM20**										

BM_b = between-species body mass; BM_w = within-species body mass; t_b = between-species time; t_w = within-species time, NC = root-to-tip node count; t_b:t_w = interaction term between both within-species time and between-species time; BM_{sp} = mean species-level body mass; t_{sp} = mean species-level time; Hnal+Hfl = dummy code distinguishing *H. naledi* and *H. floresiensis* from other hominins. Numerical values correspond to the grand means of 1,000 modelling results obtained for each one of the modelling scenarios used, excepting when percentages are reported.

Table S4, cont.: Fixed effect parameter estimates and significance for all tested models.

Part 4 of 4.

Model Code	BM_{sp}	BM_{sp} (L95CI)	BM_{sp} (U95CI)	BM_{sp} (pMCMC)	BM_{sp} (Pct < 0.05)	t_{sp}	t_{sp} (L95CI)	t_{sp} (U95CI)	t_{sp} (pMCMC)	t_{sp} (Pct < 0.05)	Hnal+Hflor	Hnal+Hflor (L95CI)	Hnal+Hflor (U95CI)	Hnal+Hflor (pMCMC)	Hnal+Hflor (Pct < 0.05)
**M1**															
**M2**															
**M3**															
**M4**															
**AM1**															
**AM2**															
**AM3**															
**AM4**															
**AM5**															
**AM6**															
**AM7**															
**AM8**															
**AM9**															
**AM10**															
**AM11**	0.812202602	0.24398823	1.385011174	0.011610606	99.50%	-4.9769E-05	-0.000101986	2.9734E-06	0.070852525	27.10%					
**AM12**															
**AM13**															
**AM14**															
**AM15**															
**AM16**															
**AM17**															
**AM18**											-0.205	-0.396	-0.014	0.061	62.50%
**AM19**															
**AM20**															

Table S5: Random effect variance estimates for all tested models.

Model Code	phy	phy (L95CI)	phy (U95CI)	slopes BM_w	slopes BM_w (L95CI)	slopes BM_w (U95CI)	slopes t_w	slopes t_w (L95CI)	slopes t_w (U95CI)	$date_{max}$	$date_{max}$ (L95CI)	$date_{max}$ (U95CI)	$date_{min}$	$date_{min}$ (L95CI)	$date_{min}$ (U95CI)	CC_{method}	CC_{method} (L95CI)	CC_{method} (U95CI)
**M1**	0.006	0.002	0.011				0.001	0.001	0.002									
**M2**	0.006	0.002	0.010	0.001	0.001	0.001	0.001	0.001	0.002									
**M3**	0.006	0.002	0.010				0.001	0.001	0.002									
**M4**	0.006	0.002	0.011															
**AM1**	0.006	0.002	0.011															
**AM2**	0.005	0.002	0.011				0.001	0.001	0.002									
**AM3**	0.007	0.002	0.012				0.001	0.001	0.002									
**AM4**	0.006	0.002	0.010				0.001	0.001	0.002									
**AM5**	0.005	0.002	0.010	0.001	0.001	0.001	0.001	0.001	0.002									
**AM6**	0.006	0.002	0.011				0.001	0.001	0.002									
**AM7**	0.006	0.002	0.011							0.004	0.001	0.008	0.004	0.001	0.008			
**AM8**	0.006	0.002	0.011	0.001	0.001	0.002				0.004	0.001	0.008	0.004	0.001	0.008			
**AM9**	0.006	0.002	0.010	0.001	0.001	0.001	0.001	0.001	0.002	0.004	0.001	0.008	0.004	0.001	0.008			
**AM10**	0.006	0.002	0.011															
**AM11**	0.006	0.002	0.011															
**AM12**	0.005	0.002	0.010				0.001	0.001	0.002									
**AM13**	0.005	0.002	0.009	0.001	0.001	0.002	0.001	0.001	0.002									
**AM14**	0.006	0.002	0.011				0.001	0.001	0.002									
**AM15**	0.006	0.002	0.011				0.001	0.001	0.002									
**AM16**	0.006	0.002	0.011				0.001	0.001	0.002							0.010	0.001	0.030
**AM17**	0.006	0.002	0.011				0.001	0.001	0.002									
**AM18**	0.005	0.002	0.008				0.001	0.001	0.002									
**AM19**	0.008	0.003	0.015				0.0014	0.0009	0.0019									
**AM20**	0.006	0.002	0.012				0.0015	0.0009	0.0021									

Dataset S1 (separate file). Temporal ranges for all species used in the tree reconstruction. Minimum and maximum dates are provided for each taxon. All sources are cited in the file.

Dataset S2 (separate file). The morphological character matrix (nexus format) used for tree construction, with n=391 characters and n=24 taxa.

Dataset S3 (separate file). The sample of 1,000 dated phylogenetic trees used for analysis.

Dataset S4 (separate file). Specimen-level data for hominins including morphology, taxonomy, temporal range, geographical location. All sources are cited in the file.

Dataset S5 (separate file). The sample of 1,000 datasets used for our analyses incorporating taxonomic, temporal, and morphological uncertainty.

SI References

1. M. Lynch, Methods for the analysis of comparative data in evolutionary biology. *Evolution* **45**, 1065–1080 (1991).

2. E. A. Housworth, E. P. Martins, M. Lynch, The phylogenetic mixed model. *The American Naturalist* **163**, 84–96 (2004).

3. J. Felsenstein, Comparative methods with sampling error and within-species variation: contrasts revisited and revised. *Am Nat* **171**, 713–725 (2008).

4. J. D. Hadfield, S. Nakagawa, General quantitative genetic methods for comparative biology: phylogenies, taxonomies and multi-trait models for continuous and categorical characters. *Journal of Evolutionary Biology* **23**, 494–508 (2010).

5. J. A. Davis, J. L. Spaeth, C. Huson, A Technique for Analyzing the Effects of Group Composition. *American Sociological Review* **26**, 215–225 (1961).

6. M. van de Pol, J. Wright, A simple method for distinguishing within- versus between-subject effects using mixed models. *Animal Behaviour* **77**, 753–758 (2009).

7. R. P. Freckleton, P. H. Harvey, M. Pagel, Phylogenetic Analysis and Comparative Data: A Test and Review of Evidence. *The American Naturalist* **160**, 712–726 (2002).

8. P. de Villemereuil, S. Nakagawa, "General Quantitative Genetic Methods for Comparative Biology" in *Modern Phylogenetic Comparative Methods and Their Application in Evolutionary Biology: Concepts and Practice*, L. Z. Garamszegi, Ed. (Springer, 2014), pp. 287–303.

9. T. F. Hansen, S. H. Orzack, Assessing Current Adaptation and Phylogenetic Inertia as Explanations of Trait Evolution: The Need for Controlled Comparisons. *evol* **59**, 2063–2072 (2005).

10. G. E. Leventhal, S. Bonhoeffer, Potential Pitfalls in Estimating Viral Load Heritability. *Trends in Microbiology* **24**, 687–698 (2016).

11. H. P. Püschel, O. C. Bertrand, J. E. O'Reilly, R. Bobe, T. A. Püschel, Divergence-time estimates for hominins provide insight into encephalization and body mass trends in human evolution. *Nat Ecol Evol* **5**, 808–819 (2021).

12. F. Ronquist, *et al.*, A total-evidence approach to dating with fossils, applied to the early radiation of the hymenoptera. *Systematic Biology* **61**, 973–999 (2012).

13. C. Zhang, T. Stadler, S. Klopfstein, T. A. Heath, F. Ronquist, Total-Evidence Dating under the Fossilized Birth-Death Process. *Systematic Biology* **65**, 228–249 (2016).

14. A. Gavryushkina, *et al.*, Bayesian total-evidence dating reveals the recent crown radiation of penguins. *Systematic Biology* **66**, 57–73 (2017).

15. S. Höhna, *et al.*, RevBayes: Bayesian Phylogenetic Inference Using Graphical Models and an Interactive Model-Specification Language. *Systematic Biology* **65**, 726–736 (2016).

16. T. Stadler, A. Gavryushkina, R. C. M. Warnock, A. J. Drummond, T. A. Heath, The fossilized birth-death model for the analysis of stratigraphic range data under different speciation modes. *Journal of Theoretical Biology* **447**, 41–55 (2018).

17. F. Bokma, V. van den Brink, T. Stadler, Unexpectedly Many Extinct Hominins. *Evolution* **66**, 2969–2974 (2012).

18. S. Katoh, *et al.*, New geological and palaeontological age constraint for the gorilla–human lineage split. *Nature* **530**, 215–218 (2016).

19. H. P. Püschel, O. C. Bertrand, J. E. O'Reilly, R. Bobe, T. A. Püschel, Divergence-time estimates for hominins provide insight into encephalization and body mass trends in human evolution. *Nature Ecology & Evolution* 1–12 (2021). https://doi.org/10.1038/s41559-021-01431-1.

20. H. P. Püschel, O. C. Bertrand, J. E. Reilly, R. Bobe, T. A. Püschel, Reply to: Modelling hominin evolution requires accurate hominin data. *Nature Ecology & Evolution* **6**, 1092–1094 (2022).

21. M. Dembo, *et al.*, The evolutionary relationships and age of Homo naledi: An assessment using dated Bayesian phylogenetic methods. *Journal of Human Evolution* **97**, 17–26 (2016).

22. Y. Haile-Selassie, S. M. Melillo, A. Vazzana, S. Benazzi, T. M. Ryan, A 3.8-million-year-old hominin cranium from Woranso-Mille, Ethiopia. *Nature* **573**, 214–219 (2019).

23. D. Argue, C. P. Groves, M. S. Y. Lee, W. L. Jungers, The affinities of Homo floresiensis based on phylogenetic analyses of cranial, dental, and postcranial characters. *Journal of Human Evolution* **107**, 107–133 (2017).

24. P. Lewis, A Likelihood Approach to Estimating Phylogeny from Discrete Morphological Character Data. *Society of Systematic Biologists* **50**, 913–925 (2001).

25. E. S. Allman, J. A. Rhodes, Identifying evolutionary trees and substitution parameters for the general Markov model with invariable sites. *Mathematical Biosciences* **211**, 18–33 (2008).

26. T. A. Heath, B. R. Moore, Bayesian inference of species divergence times. *Bayesian phylogenetics: methods, algorithms, and applications* 277–318 (2014).

27. M. Plummer, N. Best, K. Cowles, K. Vines, CODA: convergence diagnosis and output analysis for MCMC. *R news* **6**, 7–11 (2006).

28. R Core Team, R: A Language and Environment for Statistical Computing. (2022). Deposited 2022.

29. M. Will, M. Krapp, J. T. Stock, A. Manica, Different environmental variables predict body and brain size evolution in Homo. *Nat Commun* **12**, 4116 (2021).

30. M. Will, J. T. Stock, Spatial and temporal variation of body size among early Homo. *Journal of Human Evolution* **82**, 15–33 (2015).

31. S. L. Robson, B. Wood, Hominin life history: reconstruction and evolution. *Journal of Anatomy* **212**, 394–425 (2008).

32. C. B. Ruff, M. L. Burgess, N. Squyres, J.-A. Junno, E. Trinkaus, Lower limb articular scaling and body mass estimation in Pliocene and Pleistocene hominins. *Journal of Human Evolution* **115**, 85–111 (2018).

33. C. B. Ruff, N. Squyres, J.-A. Junno, Body mass estimation in hominins from humeral articular dimensions. *American Journal of Physical Anthropology* **173**, 480–499 (2020).

34. B. Wood, *Wiley-Blackwell encyclopedia of human evolution* (John Wiley & Sons, 2011).

35. D. M. Olson, *et al.*, Terrestrial Ecoregions of the World: A New Map of Life on Earth: A new global map of terrestrial ecoregions provides an innovative tool for conserving biodiversity. *BioScience* **51**, 933–938 (2001).

36. A. R. Ives, M. R. Helmus, Generalized linear mixed models for phylogenetic analyses of community structure. *Ecological Monographs* **81**, 511–525 (2011).

37. G. E. Leventhal, S. Bonhoeffer, Potential Pitfalls in Estimating Viral Load Heritability. *Trends in Microbiology* **24**, 687–698 (2016).

38. S. Nakagawa, H. Schielzeth, A general and simple method for obtaining R2 from generalized linear mixed-effects models. *Methods in Ecology and Evolution* **4**, 133–142 (2013).

39. J. D. Hadfield, E. A. Heap, F. Bayer, E. A. Mittell, N. M. A. Crouch, Intraclutch differences in egg characteristics mitigate the consequences of age-related hierarchies in a wild passerine. *Evolution* **67**, 2688–2700 (2013).

40. J. Hawks, No brain expansion in Australopithecus boisei. *American Journal of Physical Anthropology* **146**, 155–160 (2011).

41. J. Bruggeman, J. Heringa, B. W. Brandt, PhyloPars: estimation of missing parameter values using phylogeny. *Nucleic Acids Res* **37**, W179-184 (2009).

42. E. W. Goolsby, J. Bruggeman, C. Ané, Rphylopars: fast multivariate phylogenetic comparative methods for missing data and within-species variation. *Methods in Ecology and Evolution* **8**, 22–27 (2017).
